# Relative Citation Ratio (RCR): A New Metric That Uses Citation Rates to Measure Influence at the Article Level

**DOI:** 10.1371/journal.pbio.1002541

**Published:** 2016-09-06

**Authors:** B. Ian Hutchins, Xin Yuan, James M. Anderson, George M. Santangelo

**Affiliations:** 1 Office of Portfolio Analysis, National Institutes of Health, Bethesda, Maryland, United States of America; 2 Division of Program Coordination, Planning, and Strategic Initiatives, National Institutes of Health, Bethesda, Maryland, United States of America; Walter and Eliza Hall Institute of Medical Research, AUSTRALIA

## Abstract

Despite their recognized limitations, bibliometric assessments of scientific productivity have been widely adopted. We describe here an improved method to quantify the influence of a research article by making novel use of its co-citation network to field-normalize the number of citations it has received. Article citation rates are divided by an expected citation rate that is derived from performance of articles in the same field and benchmarked to a peer comparison group. The resulting Relative Citation Ratio is article level and field independent and provides an alternative to the invalid practice of using journal impact factors to identify influential papers. To illustrate one application of our method, we analyzed 88,835 articles published between 2003 and 2010 and found that the National Institutes of Health awardees who authored those papers occupy relatively stable positions of influence across all disciplines. We demonstrate that the values generated by this method strongly correlate with the opinions of subject matter experts in biomedical research and suggest that the same approach should be generally applicable to articles published in all areas of science. A beta version of *iCite*, our web tool for calculating Relative Citation Ratios of articles listed in PubMed, is available at https://icite.od.nih.gov.

## Abstract 2

A new article-level metric, the Relative Citation Ratio, provides an alternative to the use of journal impact factors as a means of identifying influential papers.

## Author Summary

Academic researchers convey their discoveries to the scientific community by publishing papers in scholarly journals. In the biomedical sciences alone, this process now generates more than one million new reports each year. The sheer volume of available information, together with the increasing specialization of many scientists, has contributed to the adoption of metrics, including journal impact factor and h-index, as signifiers of a researcher’s productivity or the significance of his or her work. Scientists and administrators agree that the use of these metrics is problematic, but in spite of this strong consensus, such judgments remain common practice, suggesting the need for a valid alternative. We describe here an improved method to quantify the influence of a research article by making novel use of its co-citation network—that is, the other papers that appear alongside it in reference lists—to field-normalize the number of times it has been cited, generating a Relative Citation Ratio (RCR). Since choosing to cite is the long-standing way in which scholars acknowledge the relevance of each other’s work, RCR can provide valuable supplemental information, either to decision makers at funding agencies or to others who seek to understand the relative outcomes of different groups of research investments.

## Introduction

In the current highly competitive pursuit of research positions and funding support [1], faculty hiring committees and grant review panels must make difficult predictions about the likelihood of future scientific success. Traditionally, these judgments have largely depended on recommendations by peers, informal interactions, and other subjective criteria. In recent years, decision makers have increasingly turned to numerical approaches such as counting first or corresponding author publications, using the impact factor of the journals in which those publications appear, and computing Hirsch (i.e., h-index) values [2]. The widespread adoption of these metrics and the recognition that they are inadequate [3–6] highlight the ongoing need for alternative methods that can provide effectively normalized and reliable data-driven input to administrative decision making, both as a means of sorting through large pools of qualified candidates and as a way to help combat implicit bias.

Though each of the above methods of quantitation has strengths, accompanying weaknesses limit their utility. Counting first or corresponding author publications does on some level reflect the extent of a scientist’s contribution to his or her field, but it has the unavoidable effect of privileging quantity over quality and may undervalue collaborative science [7]. Journal impact factor (JIF) was for a time seen as a valuable indicator of scientific quality because it serves as a convenient, and not wholly inaccurate, proxy for expert opinion [8]. However, its blanket use also camouflages large differences in the influence of individual papers. This is because impact factor is calculated as the average number of times articles published over a 2-y period in a given journal are cited; in reality, citations follow a log-normal rather than a Gaussian distribution [9]. Moreover, since practitioners in disparate fields have differential access to high-profile publication venues, impact factor is of limited use in multidisciplinary science-of-science analyses. Despite these serious flaws, JIF continues to have a large effect on funding and hiring decisions [4,10,11]. H-index, which attempts to assess the cumulative impact of the work done by an individual scientist, disadvantages early-career stage investigators; it also undervalues some fields of research by failing to normalize raw citation counts [6].

Many alternative methods for quantifying scientific accomplishment have been proposed, including citation normalization to journals or journal categories [12–19]; note that one of these is a previously described Relative Citation Rate [19], which should not be confused with the method we describe here. Other methods include citation percentiles [14,20], eigenvector normalization [21,22], and source normalization [13,23]; the latter includes both the mean normalized citation score (MNCS) [17] and source-normalized impact per paper metrics [15,17,22–26]. Although some of these methods have dramatically improved our theoretical understanding of citation dynamics [27–30], none have been widely adopted. To combine a further technical advance with a high likelihood of widespread adoption by varied stakeholders, including scientists, administrators, and funding agencies, a new citation metric must overcome several practical challenges. From a technical standpoint, a new metric must be article level, field-normalized in a way that is scalable from small to large portfolios without introducing significant bias at any level, and correlated with expert opinion. From an adoption standpoint, it should be freely accessible, calculated in a transparent fashion, and benchmarked to peer performance in a way that facilitates meaningful interpretation. Such an integrated benchmark, or comparison group, is not used by any currently available citation-based metric. Instead, all current measures aggregate articles from researchers across disparate geographical regions and institutional types, so that, for example, there is no easy way for primarily undergraduate institutions to directly compare the work they support against that of other teaching-focused institutions or for developing nations to compare their research output to that of other developing nations [31]. Enabling these and other apples-to-apples comparisons would greatly facilitate decision making by research administrators.

We report here the development and validation of the Relative Citation Ratio (RCR) metric, which is based on the novel idea of using each article’s co-citation network to field- and time-normalize the number of citations it has received; this topically linked cohort is used to derive an expected citation rate (ECR), which serves as the ratio’s denominator. As is true of other bibliometrics, article citation rate (ACR) is used as the numerator. Unlike other bibliometrics, though, RCR incorporates a customizable benchmarking feature that relates field- and time-normalized citations to the performance of a peer comparison group. RCR also meets or exceeds the standards set by other current metrics with respect to the ambitious ideals set out above. We use the RCR metric here to determine the extent to which National Institutes of Health (NIH) awardees maintain high or low levels of influence on their respective fields of research.

## Results

### Co-citation Networks Represent an Article’s Area of Influence

Choosing to cite is the long-standing way in which one scholar acknowledges the relevance of another’s work. However, the utility of citations as a metric for quantifying influence has been limited, primarily because it is difficult to compare the value of one citation to another; different fields have different citation behaviors and are composed of widely varying numbers of potential citers [32,33]. An effective citation-based evaluative tool must also take into account the length of time a paper has been available to potential citers, since a recently published article has had less time to accumulate citations than an older one. Finally, fair comparison is complicated by the fact that an author’s choice of which work to cite is not random; a widely known paper is more likely to be referenced than an obscure one of equal relevance. This is because the accrual of citations follows a power law or log-normal pattern, in accordance with a process called preferential attachment [27,33,34]. Functionally this means that, each time a paper is cited, it is a priori more likely to be cited again.

**Fig 1:**
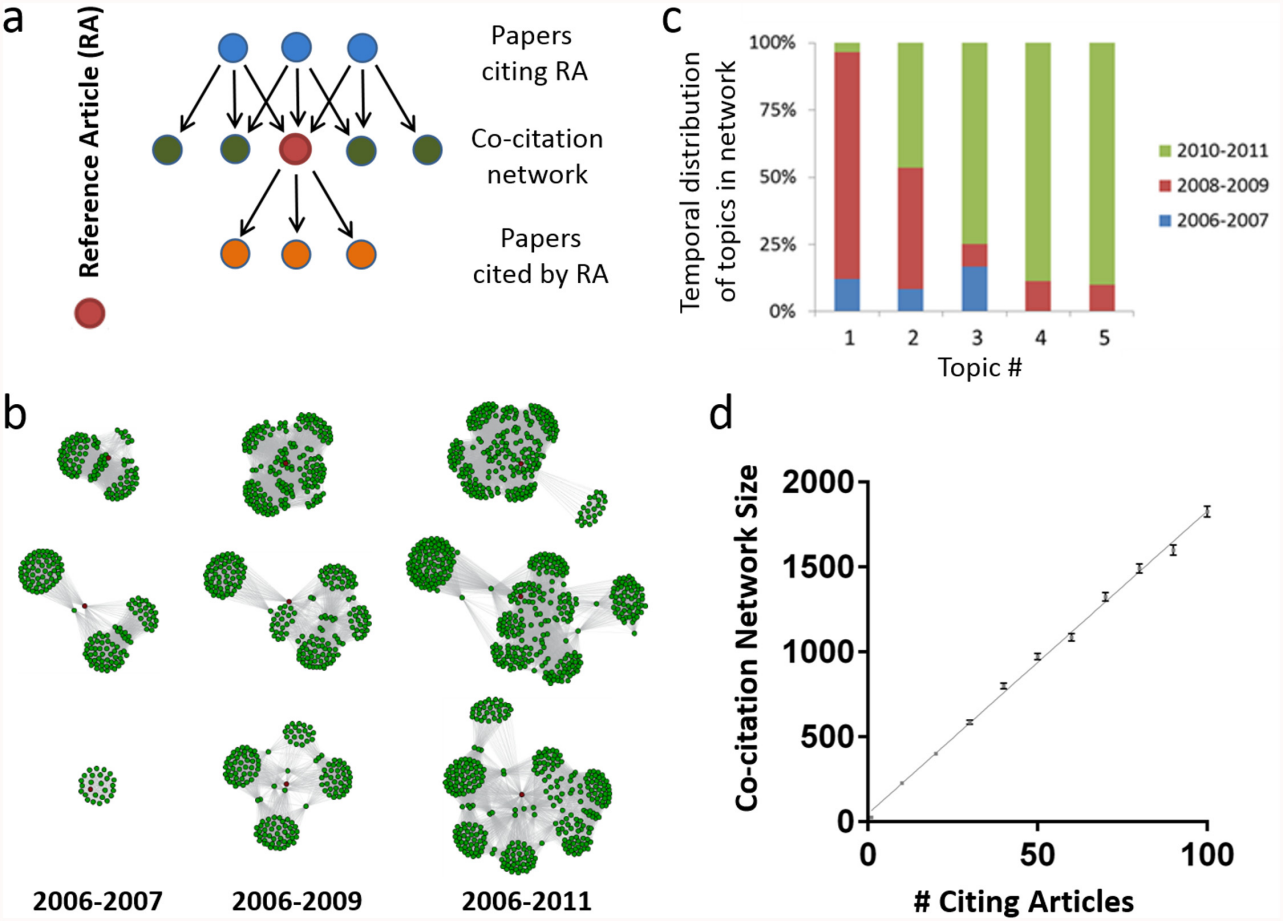
Properties of co-citation networks. (A) Schematic of a co-citation network. The reference article (RA) (red, middle row) cites previous papers from the literature (orange, bottom row); subsequent papers cite the RA (blue, top row). The co-citation network is the set of papers that appear alongside the article in the subsequent citing papers (green, middle row). The field citation rate is calculated as the mean of the latter articles’ journal citation rates. (B) Growth of co-citation networks over time. Three RAs published in 2006 (red dots) were cited 5 (top row), 9 (middle row), or 31 times (bottom row) by 2011. Three intervals were chosen to illustrate the growth of the corresponding co-citation networks: 2006–2007, 2006–2009, and 2006–2011 (the first, second, and third columns, respectively). Each article in one of the three co-citation networks is shown as a separate green dot; the edges (connections between dots) indicates their presence together in the same reference list. (C) Cluster algorithm-based content analysis of the 215 papers in the co-citation network of a sample RA (panel B, bottom network series) identified a changing pattern of relevance to different subdisciplines over time. This RA described the identification of new peptides of possible clinical utility due to their similarity to known conotoxins. Papers in the co-citation network of this RA focused on (1) α-conotoxin mechanisms of action, (2) structure and evolution of conotoxins, (3) cyclotide biochemistry, (4) conotoxin phylogenetics, and (5) identification and synthesis of lantibiotics. (D) Growth of an article’s co-citation network is proportional to the number of times it has been cited. Each point is the average network size of 1,000 randomly chosen papers with between 1 and 100 citations (error bars represent the standard error of the mean). Each paper is only counted once, even if it is co-cited with the article of interest multiple times. An average of 17.8 new papers is added to the co-citation network for each additional citation. This suggests substantial duplication of articles within a co-citation network, since on average 32.4 papers (median of 30) are referenced in each citing article.

An accurate citation-based measure of influence must address all of these issues, but we reasoned that the key to developing such a metric would be the careful identification of a comparison group, i.e., a cluster of interrelated papers against which the citation performance of an article of interest, or reference article (RA), could be evaluated. Using a network of papers linked to that RA through citations occurred to us as a promising possibility (Fig 1). There are a priori three types of article-linked citation networks [35]. A citing network is the collection of papers citing the RA (Fig 1A, top row), a co-citation network is defined as the other papers appearing in the reference lists alongside the RA (Fig 1A, middle row), and a cited network is the collection of papers in the reference list of the RA (Fig 1A, bottom row).

All three types of networks would be expected to accurately reflect the interdisciplinary nature of modern biomedical research and the expert opinion of publishing scientists, who are themselves the best judges of what constitutes a field. By leveraging this expertise, networks define empirical field boundaries that are simultaneously more flexible and more precise than those imposed by traditional bibliometric categories such as “biology and biochemistry” or “molecular biology.” An analysis of the co-citation network of a sample RA illustrates this point. The RA in the bottom panel of Fig 1B describes the identification of new peptides structurally similar to conotoxins, a little-known family of proteins that has begun to attract attention as the result of recent work describing their potential clinical utility [36]. Although the papers in this network are all highly relevant to the study of conotoxins, they cross traditional disciplinary boundaries to include such diverse fields as evolutionary biology, structural biology, biochemistry, genetics, and pharmacology (Fig 1C).

Unlike cited networks, citing and co-citation networks can grow over time, allowing for the dynamic evaluation of an article’s influence; as illustrated by the example above, they can also indicate whether or not an article gains relevance to additional disciplines (Fig 1B and 1C). An important difference between citing and co-citation networks, however, is size. Since papers in the biomedical sciences have a median of 30 articles in their reference lists, each citation event can be expected to add multiple papers to an article’s co-citation network (Fig 1D) but only one to its citing network. The latter are therefore highly vulnerable to finite number effects; in other words, for an article of interest with few citations, small changes in the citing network would have a disproportionate effect on how that article’s field was defined. We therefore chose to pursue co-citation networks as a way to describe an individual paper’s field.

**Fig 2:**
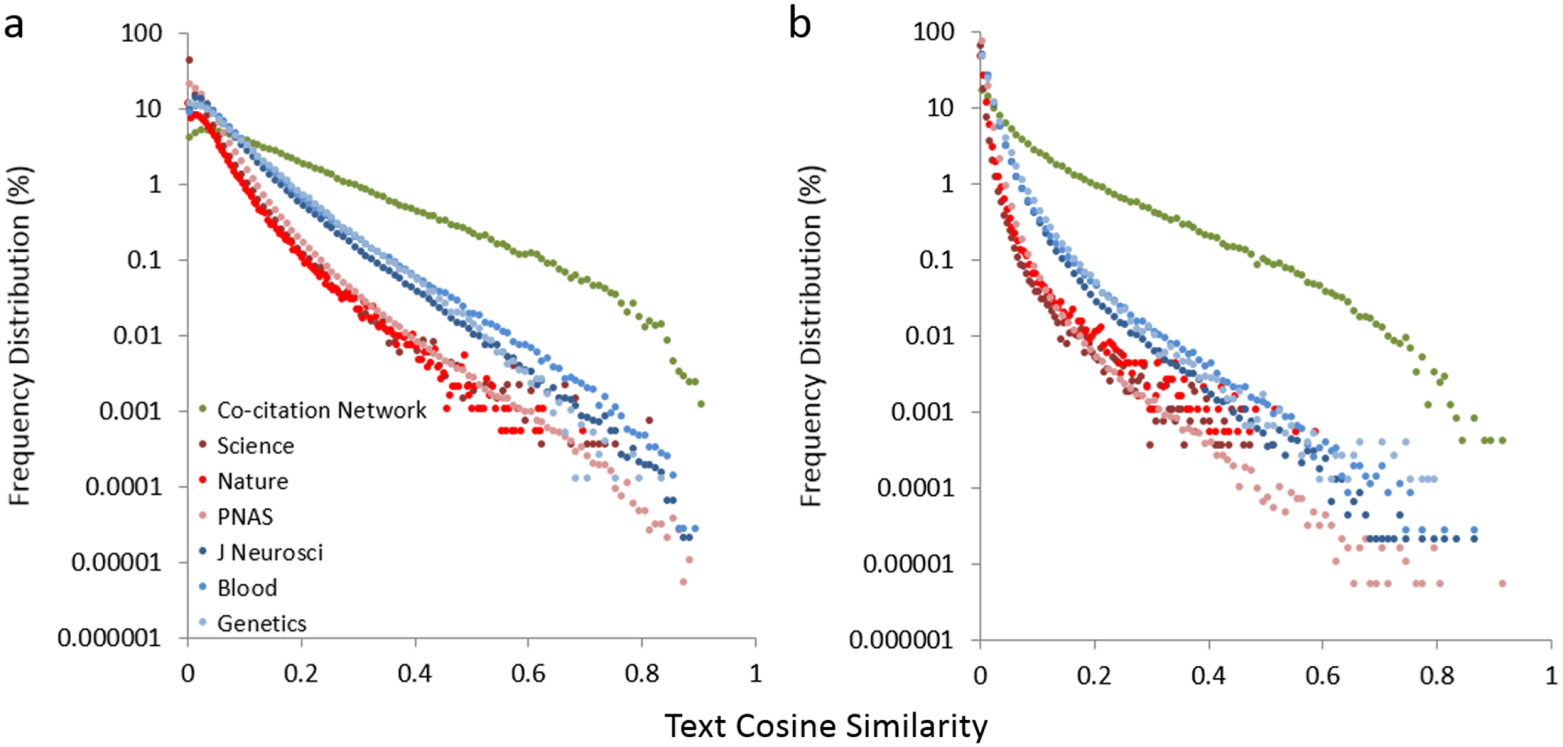
Text similarity of articles is defined more accurately by their co-citation networks than by the journals in which they appear. (A, B) The text in each of 1,397 RAs was compared, either with the text in each corresponding co-citation network or separately with the collection of articles appearing in the same journal. Both primary and review articles are included. Cosine similarity scores were then calculated using either the top 100 terms (A) or all terms appearing in at least ten documents (B). Filled circles in green, co-citation network comparison; filled circles in shades of red, multidisciplinary journal comparison; filled circles in shades of blue, disciplinary journal comparison. Curves shifted to the right show more text similarity: RAs are least similar to papers in the same multidisciplinary journals, more similar to papers in the same disciplinary journal, and most similar to papers in their co-citation network.

Having chosen our comparison group, we looked for a way to test how accurately co-citation networks represent an article’s field. One way to characterize groups of documents is to cluster them based on the frequency at which specific terms appear in a particular document relative to the frequency at which they appear in the entire corpus, a method known as term frequency–inverse document frequency (TF-IDF) [37]. This is not a perfect approach, as it is possible to use entirely different words to describe similar concepts, but positive matches can be taken as a strong indication of similarity. Frequency of word occurrence can be converted into vectors so that the cosine of the angle between two vectors is a measurement of how alike the two documents are. To evaluate co-citation networks relative to journal of publication, which is often used as a proxy for field, we selected all papers published from 2002 to 2011 in each of six different journals that received exactly five citations during that same time frame. We used cosine similarity analysis to compare the titles and abstracts of these 1,397 works to the titles and abstracts of each article in their co-citation network and then separately to those of each article in the journal in which they appeared; selecting publications with five citations was merely a concession to limit the heavy computational workload that this analysis entailed (249,981 pairwise comparisons within co-citation networks, and 28,516,576 pairwise comparisons with other articles from the same journal). Strikingly, this analysis showed that diagnostic words are much more likely to be shared between an article and the papers in its co-citation network than between that same article and the papers that appear alongside it in its journal of publication (Fig 2). As might be expected, the data in Fig 2 also indicate that articles published in disciplinary journals are more alike than articles published in multidisciplinary journals; the latter are shown as negative controls and highlight the difference in the degree of cosine similarity between an article and its co-citation network versus its journal of publication.

### Calculating the RCR

After demonstrating that co-citation networks accurately represent an article’s field, our next step was to decide how to calculate the values that numerically represent the co-citation network of each RA. The most obvious choice, averaging the citation rates of articles in the co-citation network, would also be highly vulnerable to finite number effects. We therefore chose to average the citation rates of the journals represented by the collection of articles in each co-citation network. If a journal was represented twice, its journal citation rate (JCR) was added twice when calculating the average JCR. For reasons of algorithmic parsimony we used the JCRs for the year each article in the co-citation network was published; a different choice at this step would be expected to have little if any effect, since almost all JCRs are quite stable over time (S1 Fig; S1 Table). Since a co-citation network can be reasonably thought to correspond with a RA’s area of science, the average of all JCRs in a given network can be redefined as that RA’s field citation rate (FCR).

**Fig 3:**
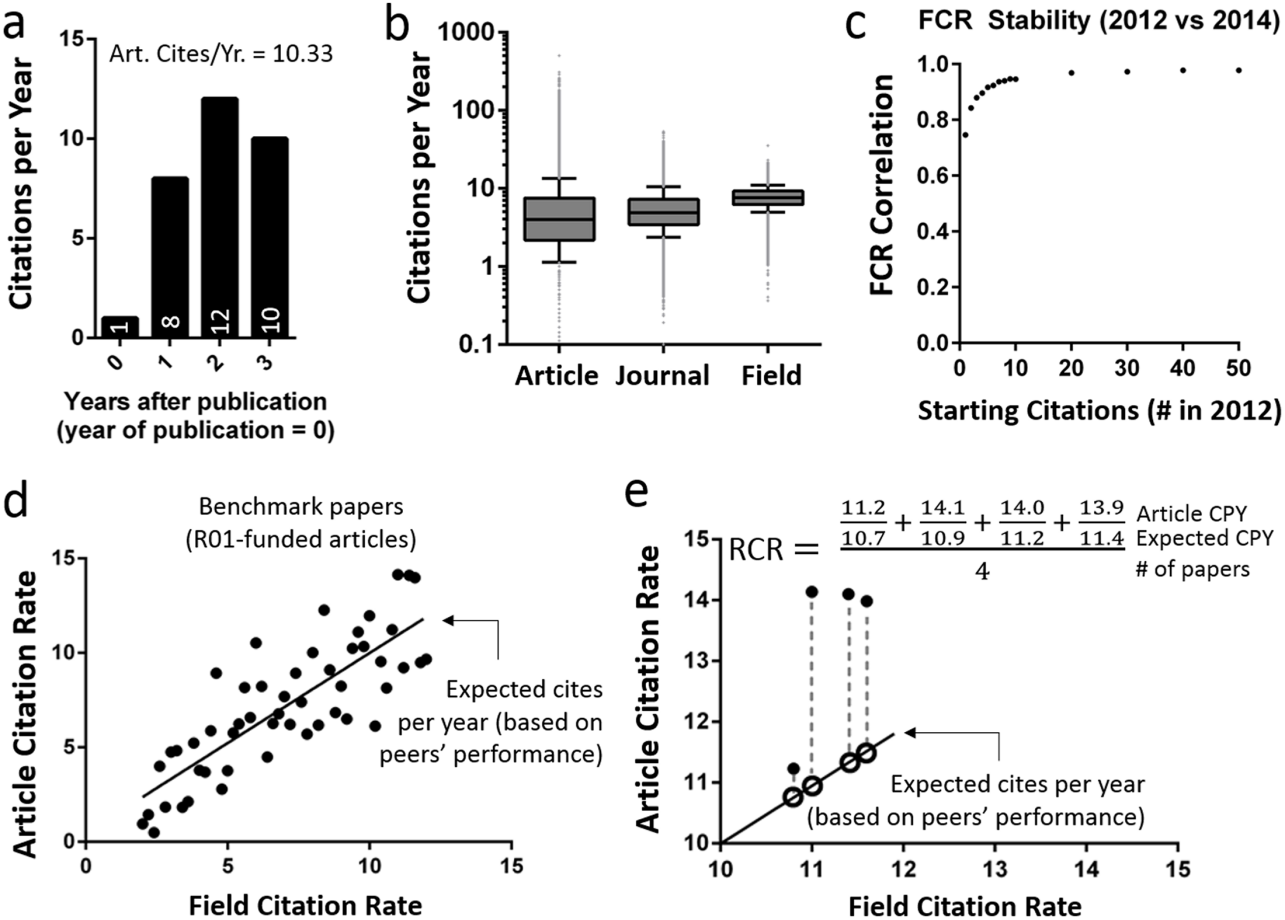
Algorithm for calculating the Relative Citation Ratio (RCR). (A) Article citation rate (ACR) is calculated as the total citations divided by the number of years excluding the calendar year of publication (Supporting Equation S1 in S1 Text), when few, if any, citations accrue (S2 Fig). Numbers in the bars correspond to the number of citations in that year. (B) Box-and-whisker plots of 88,835 NIH-funded papers (published between 2003 and 2010), summarizing their ACR, journal impact factor (matched to the article’s year of publication), and field citation rate (FCR). Boxes show the 25th–75th percentiles with a line at the median; whiskers extend to the 10th and 90th percentiles. (C) Correlation of FCR as generated in 2012 versus 2 y later in 2014 for the same set of articles, as a function of the number of starting citations in 2012. Data were sliced by the number of initial citations in 2012, to assess stability as a function of the number of citing articles (and thereby the starting size of the network). Each point, correlation coefficient for >1,000 articles. Between 2012 and 2014, articles accrued a median of 5 additional citations. The inclusion of the full span of years ensures a representative spread of ACRs at each value of the independent axis. Furthermore, since papers in this analysis receive a nearly identical number of citations in their ninth year as in their first full year after publication (S2 Fig), the FCRs of articles published later in the chosen time frame (2003 to 2010) do not undergo substantially more change than those published earlier. (D) Generate an expectation for ACRs based on a preselected benchmark group, by regressing the ACR of the benchmark papers onto their FCRs (Supporting Equations S3, S4 in S1 Text), one regression each publication year. The graphed examples were sampled from a random distribution for illustrative purposes. (E) The coefficients from each year’s regression equation transforms the FCRs of papers published in the same year into expected citation rates (ECRs) (Supporting Equation S5 in S1 Text). Each paper’s RCR is its ACR/ECR ratio. A portfolio’s RCR is simply the average of the individual articles’ RCRs (Supporting Equation S6 in S1 Text).

Using this method (Fig 3; S2 Fig; Supporting Equations S1 and S2 in S1 Text), we calculated FCRs for 35,837 papers published in 2009 by NIH grant recipients, specifically those who received R01 awards, the standard mechanism used by NIH to fund investigator-initiated research. We also calculated what the FCR would be if it were instead based on citing or cited networks. It is generally accepted that, whereas practitioners in the same field exhibit at least some variation in citation behavior, much broader variation exists among authors in different fields. The more closely a method of field definition approaches maximal separation of between-field and within-field citation behaviors, the lower its expected variance in citations per year (CPY). FCRs based on co-citation networks exhibited lower variance than those based on cited or citing networks (Table 1), suggesting that co-citation networks are better at defining an article’s field than citing or cited networks. As expected, FCRs also display less variance than either ACRs (*p* < 10^−4^, F-test for unequal variance) or JIFs (*p* < 10^−4^, F-test for unequal variance, Fig 3B, Table 1).

**Table 1:** Variance of FCRs and ECRs using different levels of the citation network for calculations (based on 35,837 R01-funded papers published in 2009).

Network Level	Variance (FCR)	Variance (ECR)
Impact factor (RA only)	33.7	30.8
Cited network	16.1	9.5
Citing network	6.7	7.8
Co-citation network	3.4	3.4

We next asked how stable the FCRs in our dataset remain over time, particularly when the starting co-citation network is small. To answer this question, we calculated FCRs for the 262,988 papers published by R01 grantees between 2003 and 2011 and cited one or more times through the end of 2012 and then recalculated FCRs for the same articles, this time using citations accrued through the end of 2014 (Fig 3C). Comparison of the two values shows that earlier FCRs are well aligned with later ones, even when the initial co-citation network was built on a single citation (Pearson correlation coefficient *r* of 0.75 versus 2 y later). The FCR quickly converged within five citations, passing *r* = 0.9 at that point (Fig 3C). The consistency that FCR values display should not be surprising, given the manner in which additional citations rapidly grow an article’s co-citation network (Fig 1D), each node of which represents a citation rate that is itself derived from a large collection of articles (Supporting Equations S1 and S2 in S1 Text). In this way, our method of calculation provides a low-variance quantitative comparator while still allowing the articles themselves to cover a highly dynamic range of subjects.

Having established the co-citation network as a means of determining an FCR for each RA, our next step was to calculate ACR/FCR ratios. Since both ACRs and FCRs are measured in CPY, this generates a rateless, timeless metric that can be used to assess the relative influence of any two RAs. However, it does not measure these values against any broader context. For example, if two RAs have ACR/FCR ratios of 0.7 and 2.1, this represents a 3-fold difference in influence, but it is unclear which of those values would be closer to the overall mean or median for a large collection of papers. One additional step is therefore needed to adjust the raw ACR/FCR ratios so that, for any given FCR, the average RCR equals 1.0. Any selected cohort of RAs can be used as a standard for anchoring expectations, i.e., as a customized benchmark (Supporting Equations S3–S6 in S1 Text). We selected R01-funded papers as our benchmark set; for any given year, regression of the ACR and FCR values of R01-funded papers yields the equation describing, for the FCR of a given RA published in that year, the ECR (Fig 3D and S2 Table). Inserting the ACR as the numerator and FCR of that RA into the regression equation as the denominator is the final step in calculating its RCR value, which incorporates the normalization both to its field of research and to the citation performance of its peers (Fig 3E and S1 Text).

We considered two possible ways to regress ACR on FCR in the benchmarking step of the RCR calculation. The ordinary least squares (OLS) approach will benchmark articles such that the mean RCR is equal to 1.0. OLS regression is suitable for large-scale analyses such as those conducted by universities or funding agencies. However, in smaller analyses in which the distribution of data may be skewed, OLS may yield an RCR less than 1.0 for the median, i.e., typical, article under consideration. In situations such as these—for example, in the case of web tools enabling search and exploration at the article or investigator level—quantile regression is more desirable, as it yields a median RCR equal to 1.0.

### Expert Validation of RCR as a Measure of Influence

**Fig 4:**
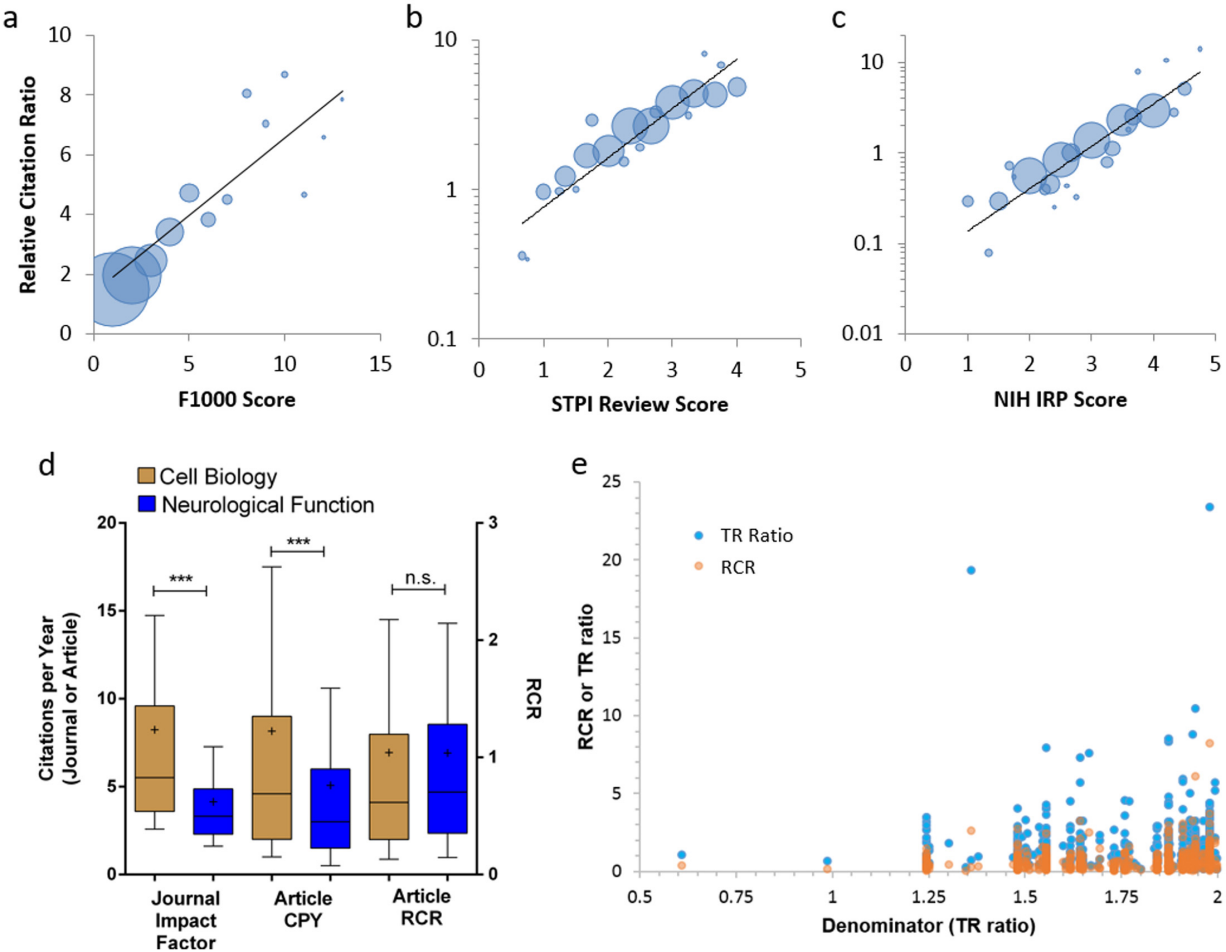
RCRs correspond with expert reviewer scores. (A–C) Bubble plots of reviewer scores versus RCR for three different datasets. Articles are binned by reviewer score; bubble area is proportionate to the number of articles in that bin. (A) F1000 scores for 2,193 R01-funded papers published in 2009. Faculty reviewers rated the articles on a scale of one to three (“good,” “very good,” and “exceptional”, respectively); those scores were summed into a composite F1000 score for each article (S3 Fig). (B) Reviewer scores of 430 HHMI and NIH-funded papers collected by STPI. (C) Scores of 290 R01-funded articles reviewed by experts from the NIH Intramural Research Program. Black line, linear regression. (D) Box-and-whisker plots illustrating the distribution of journal impact factors (JIFs) citations per year (CPY) and RCRs for two areas of NIH-funded research from 2007–2011. Cell biology, *n* = 5,936; neurological function, *n* = 5,417. *** *p* < 0.001, Kruskal-Wallis with Dunn’s multiple comparison test. n.s., not significant. Mean represented by a “+.” (E) Comparison of RCRs (orange) and Thompson Reuters ratios (blue) [17,28] for the same 544 articles with a low denominator. Data points are partially transparent to allow coordinates with multiple papers (darker) to be more clearly identified.

For the work presented here, we chose as a benchmark the full set of 311,497 RAs published from 2002 through 2012 by NIH R01 awardees. To measure the degree of correspondence between our method and expert opinion, we compared RCRs generated by OLS benchmarking of ACR/FCR values with three independent sets of postpublication evaluations by subject matter experts (details in S1 Text). We compared RCR with expert rankings (Fig 4) for 2,193 articles published in 2009 and evaluated by Faculty of 1000 members (Fig 4A and S3 Fig), as well as rankings of 430 Howard Hughes Medical Institute- or NIH-funded articles published between 2005 and 2011 and evaluated in a study conducted by the Science and Technology Policy Institute (STPI, Fig 4B and S4 Fig), and finally, 290 articles published in 2009 by extramurally funded NIH investigators and evaluated by NIH intramural investigators in a study of our own design (Fig 4C; S5, S6 and S7 Figs). All three approaches demonstrate that RCR values are well correlated with reviewers’ judgments. We asked experts in the latter study to provide, in addition to an overall score, scores for several independent subcriteria: likely impact of the research, importance of the question being addressed, robustness of the study, appropriateness of the methods, and human health relevance. Random Forest analysis [38] indicated that their scores for likely impact were weighted most heavily in determining their overall evaluation (S6 Fig).

In addition to correlating with expert opinion, RCR is ranking invariant, which is considered to be a desirable property of bibliometric indicators [39,40]. In short, an indicator is ranking invariant when it is used to place two groups of articles in hierarchical order, and the relative positions in that order do not change when uncited articles are added to each group. The RCR metric is ranking invariant when the same number of uncited articles is added to two groups of equal size (Supporting Equations S7–S9 in S1 Text). RCR is also ranking invariant when the same proportion of uncited articles is added to two groups of unequal size (Supporting Equations S10–S11 in S1 Text). This demonstrates that the RCR method can be used effectively and safely in evaluating the relative influence of large groups of publications.

### Comparison of RCR to Existing Bibliometrics

The ideal bibliometric method would provide decision makers with the means to perfectly evaluate the relative influence of even widely divergent areas of science. One way to test whether RCR represents a step towards this ambitious goal is to compare its performance in various scenarios to that of existing metrics. To begin, we asked whether RCR can fairly value a field that is disadvantaged by the use of two of the most widely recognized markers of influence: JIF and CPY. Two areas of science in which the NIH funds research are neurological function and basic cell biology. Both subjects are deserving of attention and resources; however, papers in the former field, which includes subjects such as dementia and mental health, tend to appear in lower impact factor journals and receive fewer CPY than those in the latter. In contrast, the distribution of RCR values for these two areas of study is statistically indistinguishable (Fig 4D). Although this is a single example, it does illustrate one way in which RCR provides value beyond either of these two alternative metrics.

While impact factor and CPY are two of the most commonly used evaluative measures, they are also arguably less sophisticated than other field-normalized methods advanced by bibliometricians. A recent publication has reported that RCR is better correlated with expert opinion than one of these, MNCS, but slightly less well than another, source-normalized citation score 2 (SNCS_2_) [41]. However, SNCS_2_ has an important disadvantage relative to RCR; it grows continually over time (like raw citation counts) and so is biased in favor of older papers. This disadvantage can be countered by calculating SNCS_2_ over a fixed time window, but doing so can obscure important dynamic aspects of citation behavior. In other words, if an article becomes more or less influential compared to its peers after that fixed window has passed, as for example can occur with “sleeping beauties” [42], this would not be apparent to users of SNCS_2_. These authors [41] also found that RCR is better correlated with expert opinion than citation percentiles, which is the measure bibliometricians have previously recommended as best for evaluating the impact of published work [43]. A different team of researchers has also recently reported that a simplified version of the RCR algorithm is better at identifying important papers than Google’s PageRank [44], which has previously been adapted to quantitate the influence of an article or author [45–47].

One concern that arises whenever citations are field-normalized is that papers in disciplines with intrinsically low citation rates might be inappropriately advantaged. To evaluate how RCR meets this challenge, we compared our approach to an adaptation of the MNCS method, the Thomson-Reuters (TR) ratio. Like RCR, the TR ratio uses CPY as its numerator; unlike RCR, the TR denominator is based on the average citation count of all articles published in a given year in journals that are defined as part of the same category (see S1 Text). This journal categorization approach has some problems; bibliometricians have expressed concern that it is not refined enough to be compatible with article-level metrics [48], and in the case of TR ratios, the use of proprietary journal category lists renders the calculation of the metric somewhat opaque. Still, it is a more refined measure than JIF or CPY, and like RCR, it seeks to field-normalize citations based on choice of comparison group in its denominator.

A TR ratio is available for 34,520 of the 35,837 PubMed Indexed papers published in 2009 by recipients of NIH R01 grants. For this set of articles, the TR ratio denominator ranges from 0.61 to 9.0, and a value of 2.0 or less captures 544 papers (the bottom 1.6%; Fig 4E). The average TR ratio for these papers is 1.67, and the average RCR is 0.67. Since RCR is benchmarked so that the mean paper receives a score of 1.0, it is immediately obvious that these works are having relatively little influence on their respective fields, in spite of their intrinsically low FCRs. In contrast, TR ratios are not benchmarked, so it is difficult to know whether or not it would be appropriate to flag these works as relatively low influence. Nevertheless, we can compare how each method ranks papers with these very low denominators by calculating the fraction with values above the RCR and TR ratio medians. Of the 544 low-denominator articles, 290 have a TR ratio greater than 1.07, the median value of the 34,520 papers, whereas 205 articles are above the RCR median of 0.63. This pattern holds when comparing the number of low-denominator articles that each approach places in the top 5% of the overall distribution; the journal category method identifies 17 such papers, whereas the RCR method identifies 8. Therefore, in avoiding inappropriate inflation of the assessment of articles with the lowest FCRs, RCR is at least as good as, and arguably better than, MNCS.

**Fig 5:**
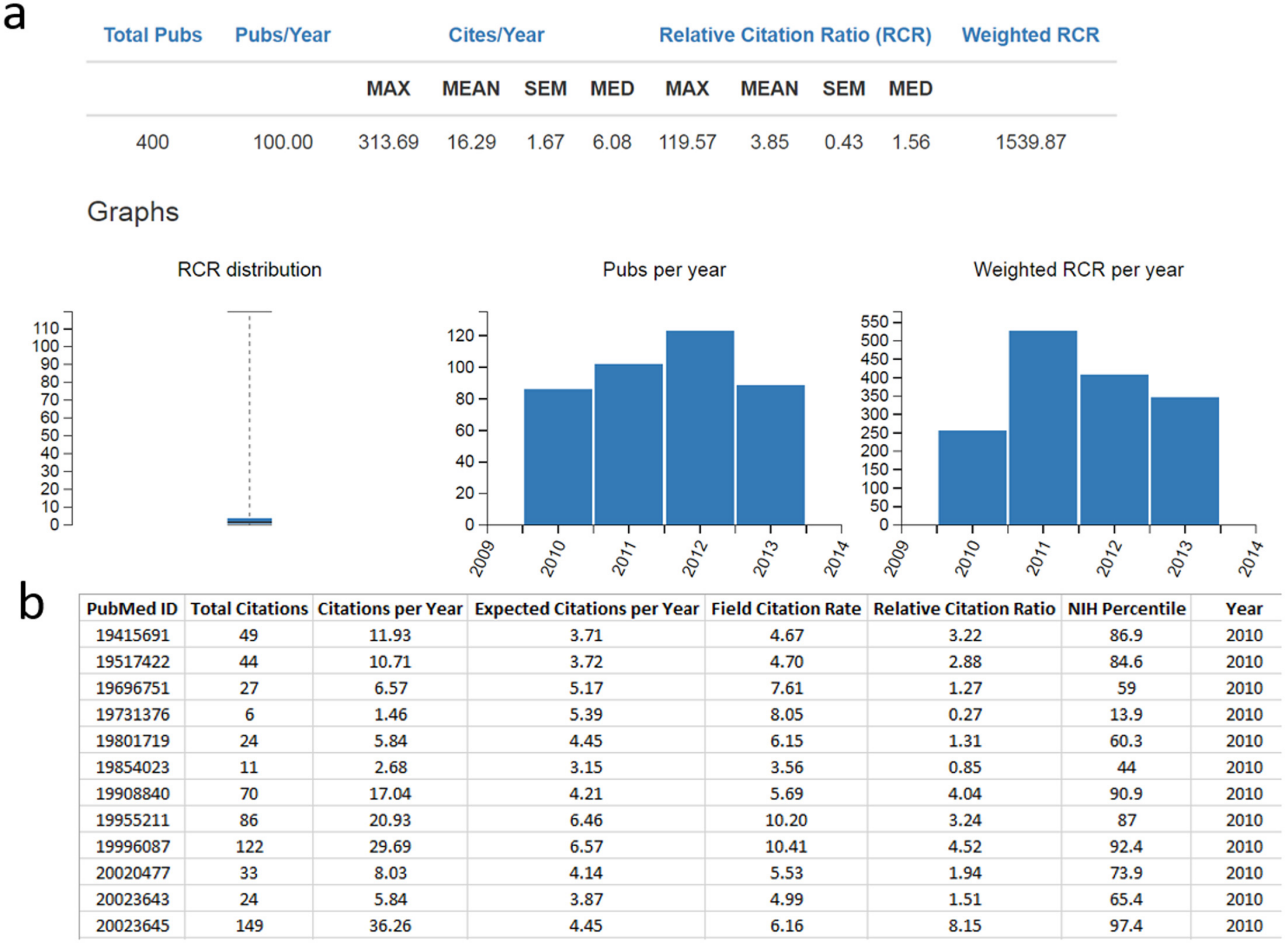
*iCite*, a publicly available tool for calculating RCR and accessing related citation information. (A) Screenshot of a sample *iCite* result. Four hundred sample PMIDs from papers published over a 4-y window were entered into the *iCite* tool, which returned the maximum, mean +/− standard error of the mean (SEM), and median values for both CPY and RCR; weighted RCR is equal to the sum of the RCRs for this group. The box-and-whisker plot shows the distribution of article RCRs; bar graphs show the number of publications per year and weighted RCR per year, respectively. (B) Sample data download for an *iCite* result. *iCite* returns the total number of citations, number of CPY, expected CPY based on an NIH benchmark, FCR, Relative Citation Ratio, and percentile ranking in a downloadable Excel format for each PMID entered, as well as the corresponding title, author information, and year/journal of publication.

Importantly, RCR is an improvement over existing metrics in terms of accessibility. While citation percentiles and TR ratios are only available through an expensive institutional subscription service, RCR values for PubMed indexed articles are freely available through the web-based *iCite* calculator, a screenshot of which is shown in Fig 5. For each PMID entered into *iCite*, users can download an Excel spreadsheet showing the total number of citations and the number of CPY received by that publication; the number of expected CPY, which are derived from a benchmark group consisting of all NIH R01 grantees, and the FCR are also reported for each article. Detailed, step-by-step help files are posted on the *iCite* website, and the full code is available on GitHub.

### RCR-Based Evaluation of Two NIH-Funded Research Programs

One of the unique strengths of RCR is the way in which a paper’s co-citation network dynamically defines its field. Each new citation an article receives, then, can be thought of as originating either from within or from outside its existing network. As a work gains relevance to additional disciplines, it seems intuitively possible that a new out-of-network citation might lead to a disproportionate increase in FCR and thus to a drop in RCR. Such an occurrence might be thought undesirable [41]; alternatively, it might be considered an accurate reflection of the reduced relative influence the work in question has on a new and larger group of scholars, many of whom previously may not have had a reason to encounter it. Regardless, we felt it was important to determine how frequently such a hypothetical scenario [41] occurs. Among the more than 200,000 articles published between 2003 and 2011 for which we calculated FCRs, only 0.2% experienced a drop in RCR of 0.1 or more between 2012 and 2014; less than 2% experienced any sort of drop at all. This low incidence is consistent with the stability we observe in FCRs (Fig 3C) and with the theoretical properties of citation networks, which are known to be scale-free and thus resistant to perturbation [49].

**Fig 6:**
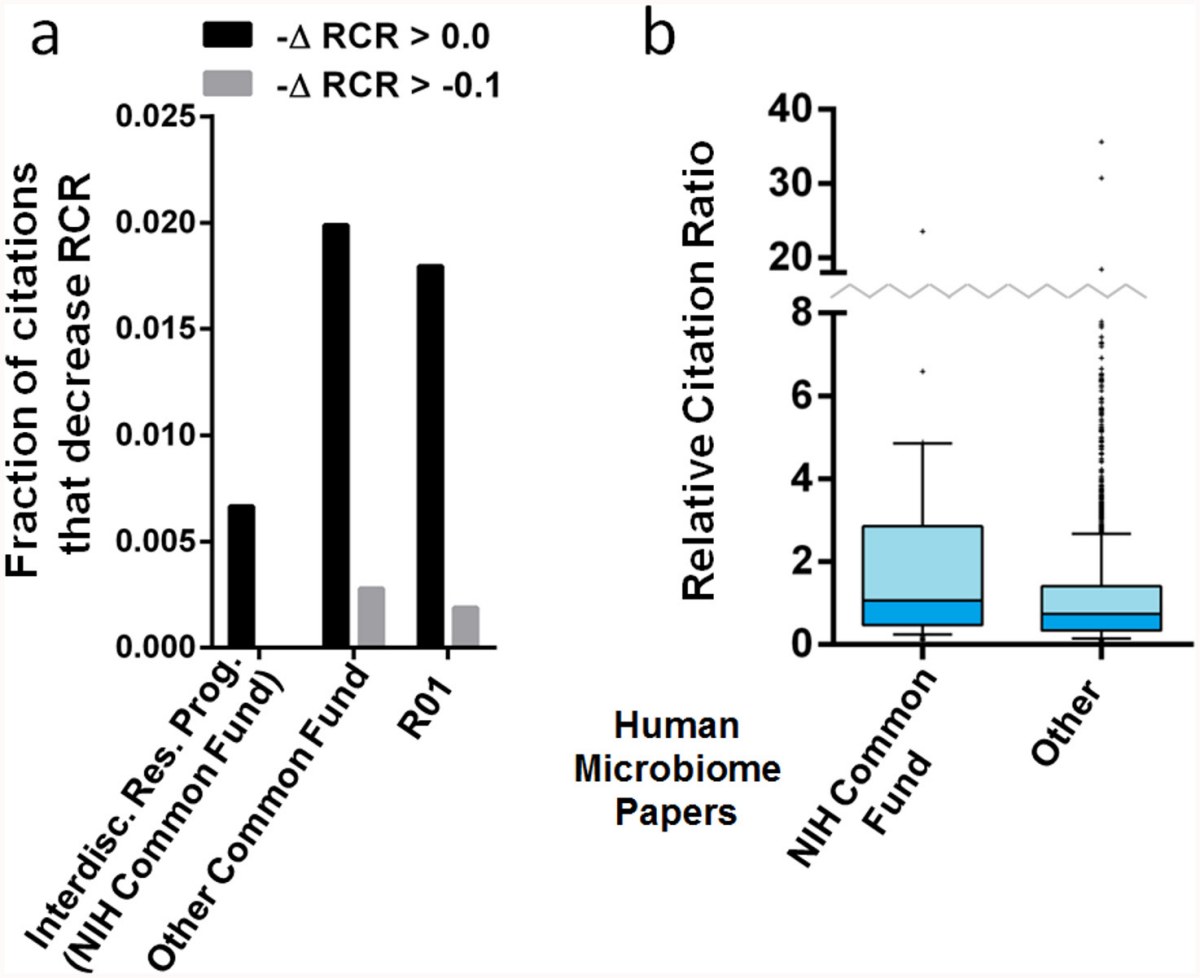
RCR-based evaluation of two NIH-funded research programs. (A) Bar graph showing the percentage of papers that experience a drop in RCR from 2012 to 2014. Black bars, decrease in RCR; grey bars, decrease in RCR of 0.1 or more. (B) Box-and-whisker plots showing the distribution of RCR values for articles describing the human microbiome, published with support from the Human Microbiome Project (HMP) of the NIH Common Fund or another source (other). Boxes show the 25th–75th percentiles with a line at the median; whiskers extend to the 10th and 90th percentiles.

While 0.2% is a very small number, we wondered whether interdisciplinary science might be overrepresented among the articles that did experience a drop in RCR. An impediment to testing this hypothesis, though, is the lack of a precisely circumscribed consensus definition of interdisciplinary research; one reason it is difficult to arrive at such a definition is that disciplines are themselves dynamic and undergo continuous evolution. For example, biochemistry may have been considered highly interdisciplinary in 1905, the year that term first appears in the PubMed indexed literature [50], but most biomedical researchers today would consider it a well-established discipline in its own right. Others might still view it as an interdisciplinary field in the strictest sense, as it occupies a space between the broader fields of biology and chemistry. To some extent, then, interdisciplinarity is in the eye of the beholder, and this presents another challenge. The question only becomes more vexed when considering more recent mergers such as computational biology, neurophysiology, or developmental genetics; are these established fields, interdisciplinary fields, or subfields? As a first approximation, then, we chose to ask whether articles produced by the NIH Interdisciplinary Research Common Fund program, which funded work that conforms to the definition of interdisciplinarity adopted by the National Academy of Sciences [51,52], were more or less likely to experience a drop in RCR than other NIH-funded articles (Fig 6). Interestingly, these interdisciplinary papers were actually 2-fold less likely to experience a drop in RCR than papers funded either by other Common Fund programs or by standard R01 support (Fig 6A). We currently lack an explanation why this might be so; given how infrequent these small drops are, we cannot yet rule out the possibility that statistical noise is responsible.

We also analyzed publications funded by NIH’s Human Microbiome Project (HMP), which was established in 2007 to generate research resources to facilitate the characterization and analysis of human microbiota in health and disease. From 2009–2011, scientists funded by the HMP published 87 articles for which citation information is available in our dataset. As a comparison group, we identified 2,267 articles on the human microbiome that were published during the same time period but were not funded by the HMP. Articles from the HMP outperformed the comparison group (Fig 6B; HMP mean RCR 2.30, median RCR 1.06; comparison RCR mean 1.23, median 0.74; *p* < 0.001, Mann-Whitney U test), demonstrating that sorting by funding mechanism has the potential to identify works of differential influence.

### Quantifying How Past Influence Predicts Future Performance

**Fig 7:**
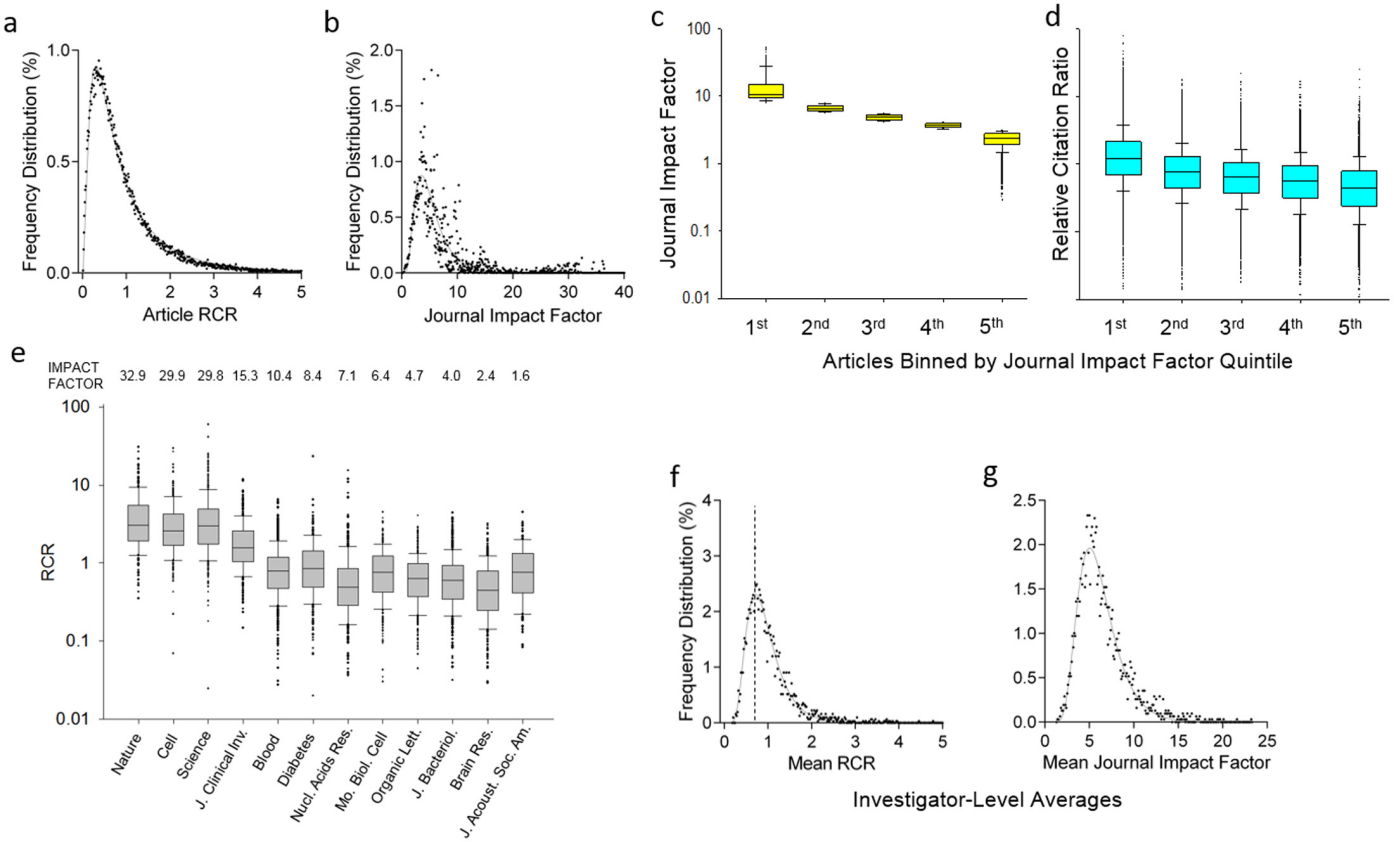
Properties of RCRs at the article and investigator level. (A, B) Frequency distribution of article-level RCRs (A) and JIFs (B), from 88,835 papers (authored by 3,089 R01-funded principal investigators [PIs]) for which co-citation networks were generated. Article RCRs are well fit by a log-normal distribution (R^2^ = 0.99), and JIFs less so (R^2^ = 0.79). (C) Box-and-whisker plots summarizing JIFs for the same papers, binned by impact factor quintile (line, median; box, 25th–75th percentiles; whiskers, 10th–90th percentiles). (D) RCR for the same papers using the same bins by JIF quintile (same scale as C). Although the median RCR for each bin generally corresponds to the impact factor quintile, there is a wide range of article RCRs in each category. (E) Box-and-whisker plots summarizing RCRs of these same papers published in selected journals. In each journal, there are papers with article RCRs surpassing the median RCR of the highest impact factor journals (left three). The impact factor of each journal is shown above. (F, G) Frequency distribution of investigator-level RCRs (F) and JIFs (G), representing the mean values for papers authored by each of 3,089 R01-funded PIs. Dashed line in (F), mode of RCR for PIs.

We next undertook a large case study of all 88,835 articles published by NIH investigators who maintained continuous R01 funding from fiscal year (FY) 2003 through FY2010 to ask how the RCR of publications from individual investigators changed over this 8-y interval. Each of these investigators had succeeded at least once in renewing one or more of their projects through the NIH competitive peer review process. In aggregate, the RCR values for these articles are well matched to a log-normal distribution (Fig 7); in contrast, as noted previously by others, the distribution of impact factors of the journals in which they were published is non-normal (Fig 7A and 7B) [53,54]. Sorting into quintiles based on JIF demonstrates that, though journals with the highest impact factors have the highest median RCR, influential publications can be found in virtually all journals (Fig 7C and 7D); these data call into question the assertion that journal of publication is the strongest quality signal for researchers who are young or otherwise unknown to a field [55]. Focusing on a dozen representative journals with a wide range of JIFs further substantiates the finding that influential science appears in many venues and reveals noteworthy departures from the correlation between JIF and median RCR (see S1 Text). For example, NIH-funded articles in both *Organic Letters* (JIF = 4.7) and the *Journal of the Acoustical Society of America* (JIF = 1.6) have a higher median RCR than those in *Nucleic Acids Research* (JIF = 7.1; Fig 7E).

**Fig 8:**
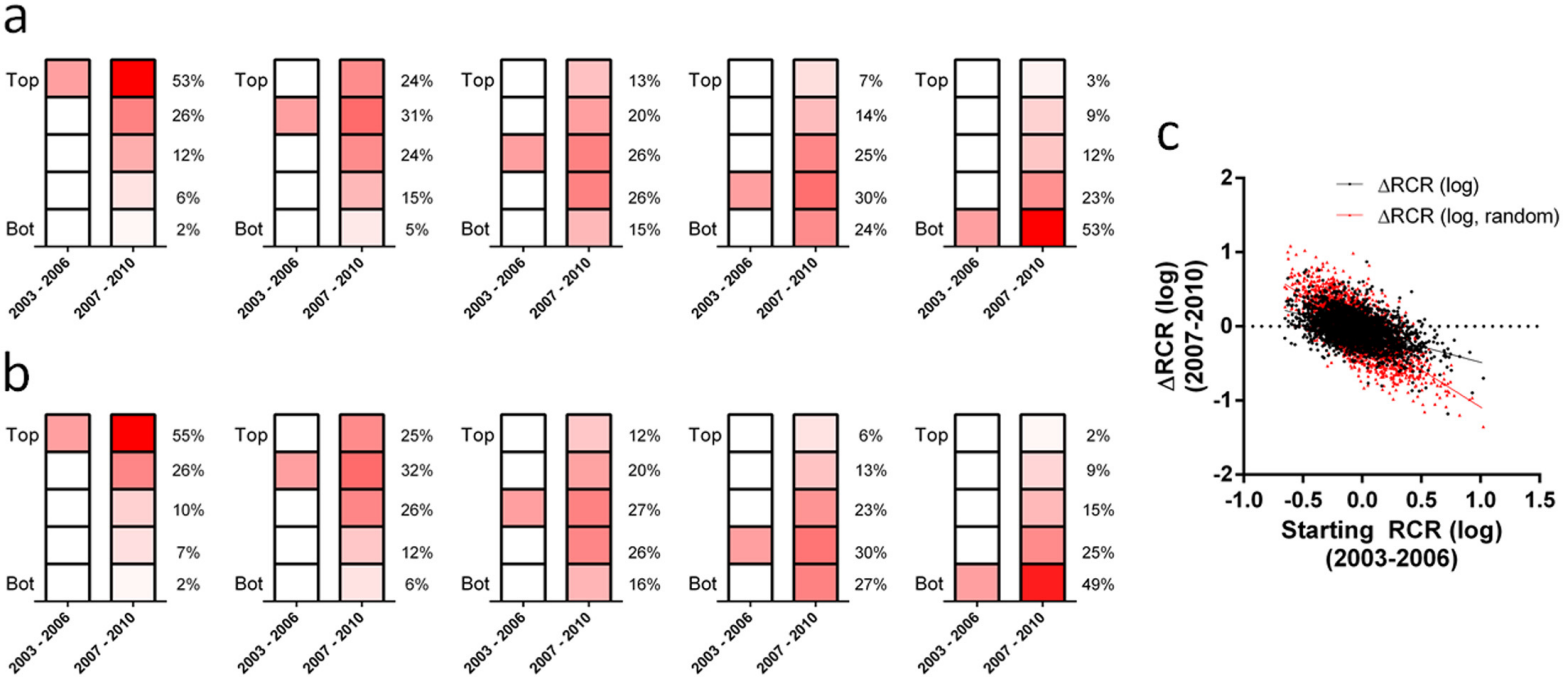
Scientific mobility of investigators’ influence relative to their field. Color intensity is proportional to the percentage of PIs in each quintile. (A) The 3,089 investigators who were continuously funded by at least one R01 were ranked by their articles’ average RCR in each time window and split into quintiles. From left to right, investigators starting in different quintiles were tracked to see their rank in the next 4-y period. (B) This panel shows the same analysis, but the number of published articles was multiplied by their average RCR to calculate an influence-weighted article count. PIs were ranked by this aggregate score and split into quintiles. (C) Scatter plot illustrating the relationship between PI RCR at earlier and later time frames. Black points, actual RCR values; black line, linear regression of actual RCR values. Red points, random assignment model (PI RCRs for the second 4-y period are reshuffled and randomly assigned); red line, linear regression of modeled data.

As part of this case study, we also calculated the average RCR and average JIF for papers published by each of the 3,089 NIH R01 principal investigators (PIs) represented in the dataset of 88,835 articles. In aggregate, the average RCR and JIF values for NIH R01 PIs exhibited log-normal distributions (Fig 7F and 7G) with substantially different hierarchical ordering (S8 Fig). This raised a further question concerning PIs with RCR values near the mode of the log-normal distribution (dashed line in Fig 7F): as measured by the ability to publish work that influences their respective fields, to what extent does their performance fluctuate? We addressed this question by dividing the 8-y window (FY2003 through FY2010) in half. Average RCRs in the first time period (FY2003 through FY2006) were sorted into quintiles, and the percentage of PIs in the second time period (FY2007 through FY2010) that remained in the same quintile, or moved to a higher or lower quintile, was calculated (Fig 8). The position of PIs in these quintiles appeared to be relatively immobile; 53% of PIs in the top quintile remained at the top, and 53% of those in the bottom quintile remained at the bottom (Fig 8A). For each PI, we also calculated a weighted RCR (the number of articles multiplied by their average RCR); comparing on this basis yielded almost identical results (Fig 8B). It is worth noting that average FCRs for investigators were extremely stable from one 4-y period to the next (Pearson *r* = 0.92, Table 2), Since FCRs are the quantitative representation of co-citation networks, this further suggests that each co-citation network is successfully capturing the corresponding investigator’s field of research.

**Table 2:** Summary of investigator-level bibliometric measures and their stability from one 4-y period to the next (PIs with more than five articles in each period, except for article count).

Measure	Mean	Median	Pearson *r* (of log-values, 2003–2006 versus 2007–2010)
Articles per Interval (PIs with >0 Articles in Both 4-y Intervals)	9.8	8.0	0.56
Field Citation Rate	7.8	7.7	0.92
Journal Citation Rate	6.3	5.7	0.76
Article Citation Rate	6.4	5.3	0.67
Relative Citation Ratio	1.0	0.85	0.61

Another possible interpretation of the above data is that PI RCRs perform an unbiased random walk from their initial state with a large diffusion rate. Considered from this frame, it could be said that 47% of PIs who started in the top quintile moved out of it during the second 4-y period we analyzed. To test this hypothesis directly, we performed a mean reversion test, which determines whether or not the set of values under consideration will return to an average, or mean, value over time. If drift in PI RCR were simply a random walk, then the change in RCR should by definition be independent of starting RCR, and plotting these two values against each other should result in a straight line with a slope of zero. However, the results show that change in RCR is dependent on starting RCR value (*p* < 0.001, linear regression analysis, *n* = 3,089, Fig 8C). Furthermore, randomly shuffling PI RCRs from the second 4-y period gives a slope that is significantly different than that observed for the real data (*p* < 0.001, extra sum-of-squares F-test, *n* = 3,089, Fig 8C), ruling out the possibility that these values are randomly sampled from the same distribution in each time interval.

## Discussion

The relationship between scientists and JIFs has been likened to the prisoner’s dilemma from game theory: because grant reviewers use JIFs in their evaluations, investigators must continue to weigh this in their decision making or risk being outcompeted by their peers on this basis [56,57]. A groundswell of support for the San Francisco Declaration on Research Assessment (http://www.ascb.org/dora) has not yet been sufficient to break this cycle [56–61]. Continued use of the JIF as an evaluation metric will fail to credit researchers for publishing highly influential work. Articles in high-profile journals have average RCRs of approximately 3. However, high-impact-factor journals (JIF ≥ 28) only account for 11% of papers that have an RCR of 3 or above. Using impact factors to credit influential work therefore means overlooking 89% of similarly influential papers published in less prestigious venues.

Bibliometrics like JIF and h-index are attractive because citations are affirmations of the spread of knowledge amongst publishing scientists and are important indicators of the influence of a particular set of ideas. Though tracking the productivity of individual scientists with bibliometrics has been controversial, it is difficult to contradict the assertion that uncited articles (RCR = 0) have little if any influence on their respective fields or that the best-cited articles (RCR > 20) are impressively influential. We have not determined whether smaller differences, for example, those with average or slightly above-average RCRs (e.g., 1.0 versus 1.2), reliably reflect differential levels of influence. Further, citation-based metrics can never fully capture all of the relevant information about an article, such as the underlying value of a study or the importance of making progress in solving the problem being addressed. The RCR metric is also not designed to be an indicator of long-term impact, and citation metrics are not appropriate for applied research, e.g., work that is intended to target a narrow audience of nonacademic engineers or clinicians.

It is also very important to note that like all other citation-based metrics, an RCR value cannot be calculated immediately after an article is published. Instead, enough time must pass for a meaningful number of citations to accrue, and the work we describe here provides some rough guidance as to what that meaningful number might be. Specifically, we have found that 93% of co-citation network-based FCRs stabilize after a work has been cited five times (Fig 3C); also in agreement with previously published work, citation rates for approximately the same percentage of articles peak within 2 to 3 y after publication (S2 Fig). Before one or both of those benchmarks have been reached, RCR values might be viewed as provisional; even after that point, neither RCR nor any other citation-based metric should be taken as a substitute for the actual reading of a paper in determining its quality. However, as citation rates mark the breadth and speed of the diffusion of knowledge among publishing scholars, we maintain that quantitative metrics based on citations can effectively supplement subject matter expertise in the evaluation of research groups seeking to make new discoveries and widely disseminate their findings.

We believe RCR offers some significant advantages over existing citation-based metrics, both technically and in terms of usability. Technically, prior attempts to describe a normalized citation metric have resulted in imperfect systems for the comparison of diverse scholarly works (S1 Text and Fig 4E), either because they measure only the average performance of a group of papers [62] or because the article of interest is measured against a control group that includes widely varying areas of science [17,32,48]. An example of the latter is citation percentiling, which the Leiden manifesto [43] recently recommended as a best practice in bibliometrics. Theoretically, the RCR method is an improvement over the use of citation percentiling alone, since masking the skewed distribution of citations and article influence, while statistically convenient, can disadvantage portfolios of high-risk, high-reward research that would be expected to have a small proportion of highly influential articles [63]. Furthermore, we have shown that co-citation networks better define an article’s field than journal of publication (Fig 2), so RCR is a more precise measure of influence than journal-based metrics, a category that includes both citation percentiles and MNCS methods such as the TR ratio. RCR is less likely to unfairly advantage publications in fields with a low citation rate than the TR ratio (Fig 4E). Finally, by incorporating a way to benchmark to a meaningful comparison group, RCR makes it easy for users to know whether a set of articles is above or below expectations for their region or agency; the need for such a feature has already been prominently discussed [31].

In terms of usability, both RCR values and their component variables, including FCRs, CPY, and total citations, are freely available to the public through our *iCite* tool. As many of the source citation data are proprietary, we are prevented from identifying all of the citing papers; presently, all bibliometrics face this challenge, as limited open source citation data are available. We feel that RCR and *iCite* represent a large improvement in transparency relative to citation percentiles and TR ratios, which are not cost-free and are furthermore dependent on the proprietary classification of journals into one or another area of science. Our method and tool are also far more transparent than impact factor, the calculation of which has recently come under scrutiny after allegations of manipulation [58,64,65].

Any metric can be gamed, and we have thought carefully about how a single author might try to game RCR. ACRs could be inflated through a combination of self-citation and frequent publication; this strategy has its limits, though, as the top 10% of RCR values for NIH-funded publications on average receive more than 25 CPY, and it is rare for a biomedical scientist to publish more than four or five times over that period. A more promising strategy might be to strive for the lowest possible FCR. An author taking this approach would need to stack the reference section of his or her work not just with poorly cited articles, or with articles in poorly cited fields, but with articles that are co-cited with articles in poorly cited fields. Since citing behavior is also constrained by content, this might be difficult to accomplish; at the very least, it seems likely that reviewers and editors would be able to identify the resulting reference list as unusual. Of course, if enough authors start to reference works in poorly cited areas, that field’s citation rate will go up, and the RCR of the papers in it may go down; in that respect, efforts to game RCR might ultimately prove to be self-defeating.

An important point to keep in mind when interpreting RCR values, though, is that citations follow a power law or log-normal distribution [9], wherein one researcher’s choice of a particular RA is at least partly informed by the choices that other researchers have previously made. There is a certain amount of noise inherent in that selection process [27], especially in the early days of a new discovery when a field is actively working towards consensus. The results of a landmark study on the relationship between quality and success in a competitive market suggest that the ultimate winners in such contests are determined not only by the intrinsic value of the work but also by more intangible social variables [66]. Consistent with this conclusion, a different group of authors has shown that including a “reputation” variable enables an algorithm to better predict which papers in the interdisciplinary field of econophysics will be the most highly cited [67]. Although there is on average a positive relationship between quality and success [66], it is for this reason we suggest that RCR should primarily be considered as a measure of influence, rather than impact or intellectual rigor.

Within these bounds, bibliometric methods such as RCR have the potential to track patterns of scientific productivity over time, which may help answer important questions about how science progresses. In particular, co-citation networks can be used to characterize the relationship between scientific topics (including interdisciplinarity), emerging areas, and social interactions. For example, is the membership of an influential group of investigators in a given field or group of fields stable over time, or is it dynamic, and why? Our data demonstrate the existence of an established hierarchy of influence within the exclusive cohort of NIH R01 recipients who remained continuously funded over an 8-y time frame. This may mean that investigators tend to ask and answer questions of similar interest to their fields. Additionally or alternatively, stable differences in investigators’ status, such as scientific pedigree, institutional resources, and/or peer networks, may be significant drivers of persistently higher or lower RCR values. Future statistical analyses may therefore reveal parameters that contribute to scholarly influence. To the extent that scientific (im)mobility is a product of uneven opportunities afforded to investigators, there may be practical ways in which funding agencies can make policy changes that increase mobility and seed breakthroughs more widely.

There is increasing interest from the public in the outcomes of research. It is therefore becoming necessary to demonstrate outcomes at all levels of funding entities’ research portfolios, beyond the reporting of success stories that can be quickly and succinctly communicated. For this reason, quantitative metrics are likely to become more prominent in research evaluation, especially in large-scale program and policy evaluations. Questions about how to advance science most effectively within the constraints of limited funding require that we apply scientific approaches to determine how science is funded [68–71]. Since quantitative analysis will likely play an increasingly prominent role going forward, it is critical that the scientific community accept only approaches and metrics that are demonstrably valid, vetted, and transparent and insist on their use only in a broader context that includes interpretation by subject matter experts. Widespread adoption of this or any other new metric should follow, not precede, extensive testing in a wide variety of real-world circumstances. Further, the RCR method was designed to assess neither the productivity of individual researchers nor the quality of their science.

Recent work has improved our theoretical understanding of citation dynamics [27–29]. However, progress in solving scientific challenges, rather than citation counts, is the primary interest of funding agencies. The NIH particularly values work that ultimately culminates in advances to human health, a process that has historically taken decades [72]. Here too, metrics have facilitated quantitation of the diffusion of knowledge from basic research toward human health studies, by examining the type rather than the count of citing articles [73]. Insights into how to accelerate this process will probably come from quantitative analysis. To credit the impact of research that may currently be underappreciated, comprehensive evaluation of funding outputs will need to incorporate metrics that can capture many other types of outputs, outcomes, and impact, such as the value of innovation, clinical outcomes, new software, patents, and economic activity. As such, the metric described here should be viewed not as a tool to be used as a primary criterion in funding decisions but as one of several metrics that can provide assistance to decision makers at funding agencies or in other situations in which quantitation can be used judiciously to supplement, not substitute for, expert opinion.

## Materials and Methods

### Citation Data

The Thomson Reuters Web of Science citation dataset from 2002–2012 was used for citation analyses. For FCR stability analysis, this dataset was extended to include 2014 data. Because of our primary interest in biomedical research, we limited our analysis to those journals in which NIH R01-funded researchers published during this time. For assigning a JCR to a published article, we used the 2-y synchronous JCR [39,74] for its journal in the year of its publication. Publications from the final year of our dataset (2012) were not included in analyses because they did not have time to accrue enough citations from which to draw meaningful conclusions, but references from these papers to earlier ones were included in citation counts. For analysis of the stability of FCRs in Fig 3, the Web of Science dataset was extended to include the years 2002–2014.

### Grant and PI Data

Grant data were downloaded from the NIH RePORTER database (https://projectreporter.nih.gov/). Grant-to-publication linkages were first derived from the NIH SPIRES database, and the data were cleaned to address false positives and false negatives. Grant and publication linkages to PIs were established using Person Profile IDs from the NIH IMPAC-II database. To generate a list of continuously funded investigators, only those Person Profile IDs with active R01 support in each year of FY2003–FY2010 were included.

### Calculations and Data Visualization

Co-citation networks were generated in Python (Python Software Foundation, Beaverton, Oregon). This was accomplished on a paper-by-paper basis by assembling the list of articles citing the article of interest and then assembling a list of each paper that those cited. This list of co-cited papers was deduplicated at this point. Example code for generating co-citation networks and calculating FCRs is available on GitHub (http://github.com/NIHOPA). Data that were used for analysis can be found as csv files in the same repository. Further calculations were handled in R (R Foundation for Statistical Computing, Vienna, Austria). Visualizations were generated in Prism 6 (GraphPad, La Jolla, California), SigmaPlot (Systat Software, San Jose, California), or Excel 2010 (Microsoft, Redmond, Washington). Code used to generate the database used in the *iCite* web application (https://icite.od.nih.gov) can be found in the GitHub repository. A preprint version of this manuscript can also be found on bioRxiv [75]. For box-and-whisker plots, boxes represent the interquartile range with a line in between at the median, and whiskers extend to the 10th and 90th percentiles.

When comparing citations rates to other metrics (e.g., postpublication review scores), citation rates were log-transformed because of their highly skewed distribution, unless these other scores were similarly skewed (i.e., Faculty of 1000 review scores). For this process, article RCRs of zero were converted to the first power of 10 lower than the lowest positive number in the dataset (generally 10^−2^). In the analysis of PI RCRs, no investigator had an average RCR of zero.

### Content Analysis

The commercially available text mining program IN-SPIRE (Pacific Northwest National Laboratories, Richland, Washington) [76] was used for content-based clustering of citations (Fig 1). For comparison of JIF CPY and RCR (Fig 4D), papers in the fields of cell biology and neurological function were those supported by grants assigned to the corresponding review units within the NIH Center for Scientific Review. For the data in Fig 2, articles were selected from six journals; three of these were disciplinary (*Journal of Neuroscience*, *Blood*, and *Genetics*), and the other three were multidisciplinary (*Nature*, *Science*, and *PNAS*). Articles published between 2002 and 2011 that accrued exactly 5 citations during that same time frame (*n* = 1,397) were selected; this subset, rather than all articles published in these six journals, was chosen for analysis in order to limit the number of pairwise text comparisons. Cosine similarity scores [37] were calculated for these 1,397 RAs against each article in their co-citation network and separately against each article appearing in the same journal. This resulted in 249,981 pairwise comparisons with articles in the co-citation networks and 28,516,576 pairwise comparisons with articles from the same journals. The journal comparisons and the co-citation comparison were both done with primary articles as well as reviews. For the comparisons, abstracts (from PubMed) were concatenated with titles to comprise the information for each document. Words were converted to lower case and stemmed. Any numbers, as well as words consisting of one or two letters, were removed from the corpus along with words appearing less than ten times. Term-document matrices were weighted for TF-IDF [77]. For one analysis, the term-document matrix was trimmed to the top 1000 TF-IDF-weighted terms, and in the other analysis, no additional term trimming was performed.

## Supporting Information

S1 Fig JIF stability over time. (A) JIFs for 12 selected journals from 2003 to 2011. (B) Pearson correlation coefficients *r* of the JIFs for these 12 journals in 2003 versus each of their respective impact factors in subsequent years. In each case, *r* is over 0.9.
(TIF)

S2 Fig Mean citations accrued each year for 608,058 papers published in 2003 appearing in the same journals as NIH-funded publications. Adding the values for 2003 and 2004 gives a value (2.26 citations per publication per year) close to the mean CPY of the following years (2.36). Although these values may seem low, they are both similar to the global 2013 Aggregate impact factor metric for journals appearing in the Biology subcategory (2.56), which is also measured in citations per paper per year.
(TIF)

S3 Fig Distribution of Faculty of 1000 scores for 2,193 R01-funded papers from 2009. (TIF)

S4 Fig Distribution of scores from the STPI survey. (TIF)

S5 Fig Summary of NIH Intramural Research Program (IRP) reviewer responses to postpublication peer review questions. Distribution of ratings to the following questions: (A) rate whether the question being addressed is important to answer; (B) rate whether you agree that the methods are appropriate and the scope of the experiments adequate; (C) rate how robust the study is based on the strength of the evidence presented; (D) rate the likelihood that the results could ultimately have a substantial positive impact on human health outcomes; (E) rate the impact that the research is likely to have or has already had; and (F) provide your overall evaluation of the value and impact of this publication.
(TIF)

S6 Fig Inferred criteria used by NIH IRP reviewers to rate overall value and impact of a publication. (A) Criteria most strongly linked to assessments of “overall value,” measured with Random Forest classification. Values indicate the mean decrease in Gini coefficient. (B) Criteria most strongly linked to assessments of “overall value,” excluding “likely impact,” and measured with Random Forest classification.
(TIF)

S7 Fig The internal correlation of postpublication peer review scores is similar to the correlation between RCR and review scores. (A) Pearson correlation coefficients (*r*) of one randomly chosen reviewer score versus the mean of the other two scores for that paper, determined by statistical resampling. Distribution of correlation coefficients determined by resampling from the STPI dataset (10,000 repetitions, mean *r* = 0.32). (B) Pearson correlation coefficients (*r*) of one randomly chosen reviewer score versus the mean of the other two scores for that paper, determined by statistical resampling. Distribution of correlation coefficients determined by resampling from the IRP dataset (10,000 repetitions, mean *r* = 0.44).
(TIF)

S8 Fig Correlation of the average of different investigators’ article RCRs versus the average of the JIFs in which they published. Some investigators published very influential articles (high RCR) in lower-profile venues (low JIF) and vice versa. R^2^ = 0.23.
(TIF)

S9 Fig Frequency distribution of the FCRs of 35,813 R01-funded articles published in 2009, using the standard approach for calculating FCRs (mean, blue) given the JCRs of the articles in the co-citation network or using the median of these JCRs to calculate the FCR (median, orange). (TIF)

S10 Fig Using mean versus median values to calculate the RCR denominator. The method for aggregating the JCRs of the articles in the co-citation network was varied and the ACR:FCR ratios compared.
(TIF)

S1 Table JIF stability over time for 100 selected journals. (XLSX)

S2 Table Regression coefficients (ACR on FCR, through 2012), for OLS linear regression or quantile regression (QR) to the median for papers with concurrent R01 funding. One of the criticisms about impact factor is that it uses the mean, rather than median, of a skewed distribution [78]; for RCR, QR can be used to benchmark articles to the median citation performance in the benchmark group, rather than the mean.
(XLSX)

S3 Table Correlation coefficients (*r*) between the log-transformed RCR values generated with the three different methods for estimating FCRs with co-citation networks. (XLSX)

S4 Table Correlation between ACR versus ECR. (XLSX)

S5 Table Effects of an attempt to game the denominator by introducing low Impact-Factor articles (40 articles of impact factor 1.0) to the co-citation network to a real article close to the average RCR. (XLSX)

S6 Table Effects on the CPY:FCR ratio of a field with a citation rate that drifts by a factor of 1.5 over the course of 10 y. (XLSX)

S7 Table Summary of investigator-level bibliometric measures and their stability from two 2-y periods spanning a decade (2002–2003 and 2012–2013, PIs with more than five articles in each period). (XLSX)

S1 Text Supporting text and equations. (DOCX)

## Abbreviations

ACR: article citation rate
CPY: citations per year
ECR: expected citation rate
FCR: field citation rate
FY: fiscal year
HMP: Human Microbiome Project
IRP: Intramural Research Program
JCR: journal citation rate
JIF: journal impact factor
MNCS: mean normalized citation score
NIH: National Institutes of Health
OLS: ordinary least squares
PI: principal investigator
QR: quantile regression
RA: reference article
RCR: Relative Citation Ratio
STPI: Science and Technology Policy Institute
TF-IDF: term frequency–inverse document frequency
TR: Thomson Reuters
