# Supplementary figures and images for: Relative Citation Ratio (RCR): A New Metric That Uses Citation Rates to Measure Influence at the Article Level
Source: PLoS Biol. 2016 Sep 6;14(9):e1002541. doi: 10.1371/journal.pbio.1002541 (PMC5012559; doi:10.1371/journal.pbio.1002541)

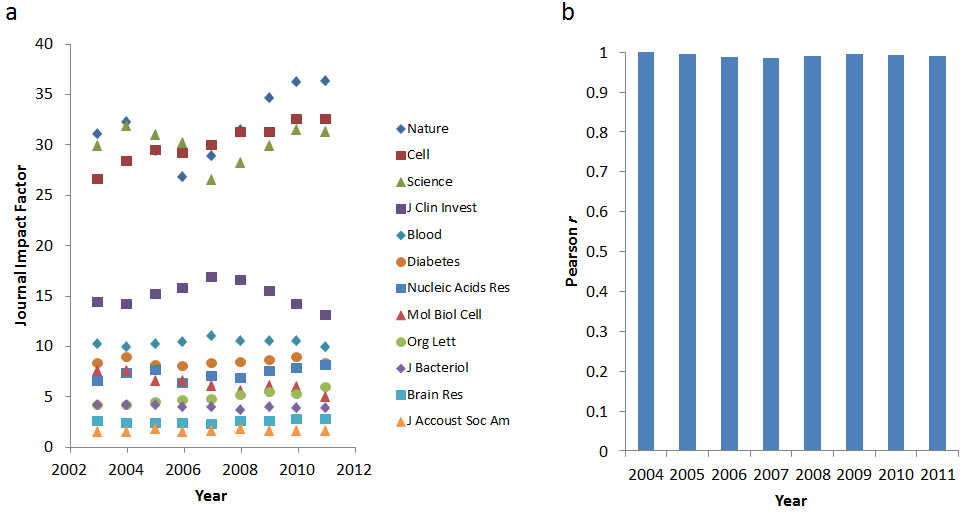

Supplement: S1 Fig — (A) JIFs for 12 selected journals from 2003 to 2011. (B) Pearson correlation coefficients r of the JIFs for these 12 journals in 2003 versus each of their respective impact factors in subsequent years. In each case, r is over 0.9. (TIF) [file pbio.1002541.s001.tif]

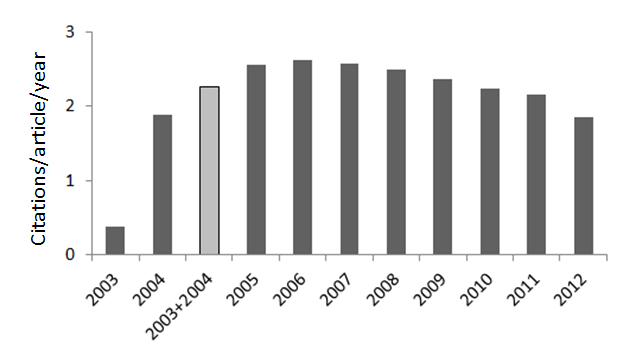

Supplement: S2 Fig — Adding the values for 2003 and 2004 gives a value (2.26 citations per publication per year) close to the mean CPY of the following years (2.36). Although these values may seem low, they are both similar to the global 2013 Aggregate impact factor metric for journals appearing in the Biology subcategory (2.56), which is also measured in citations per paper per year. (TIF) [file pbio.1002541.s002.tif]

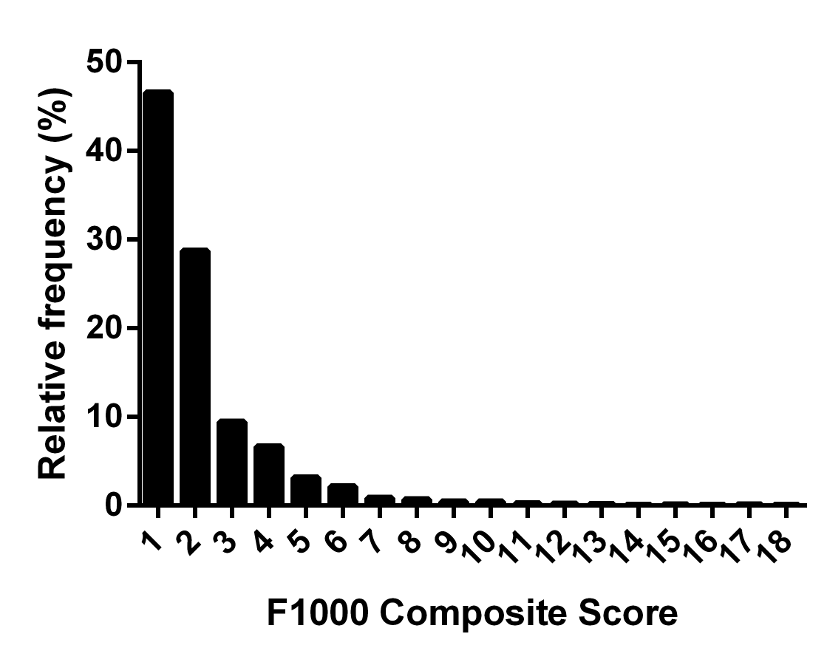

Supplement: S3 Fig — (TIF) [file pbio.1002541.s003.tif]

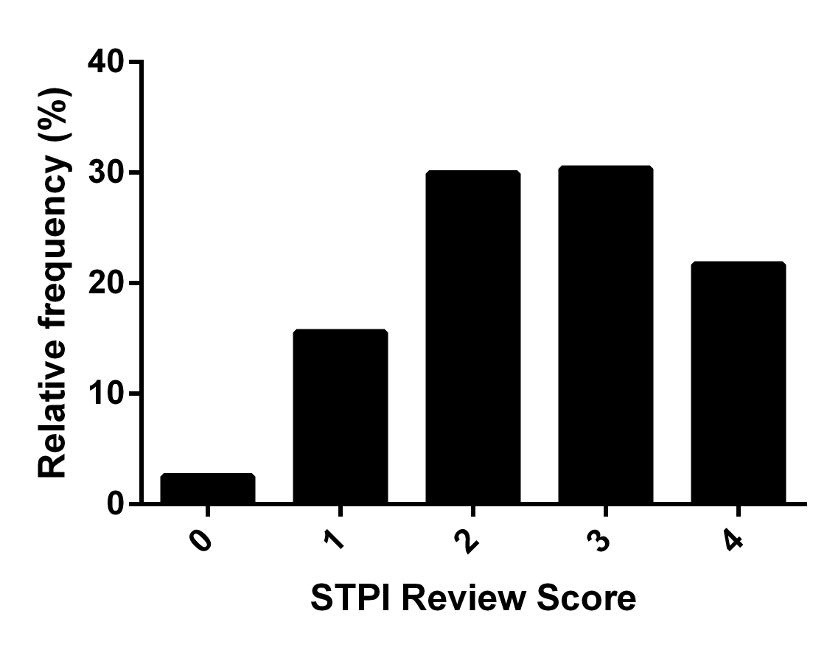

Supplement: S4 Fig — (TIF) [file pbio.1002541.s004.tif]

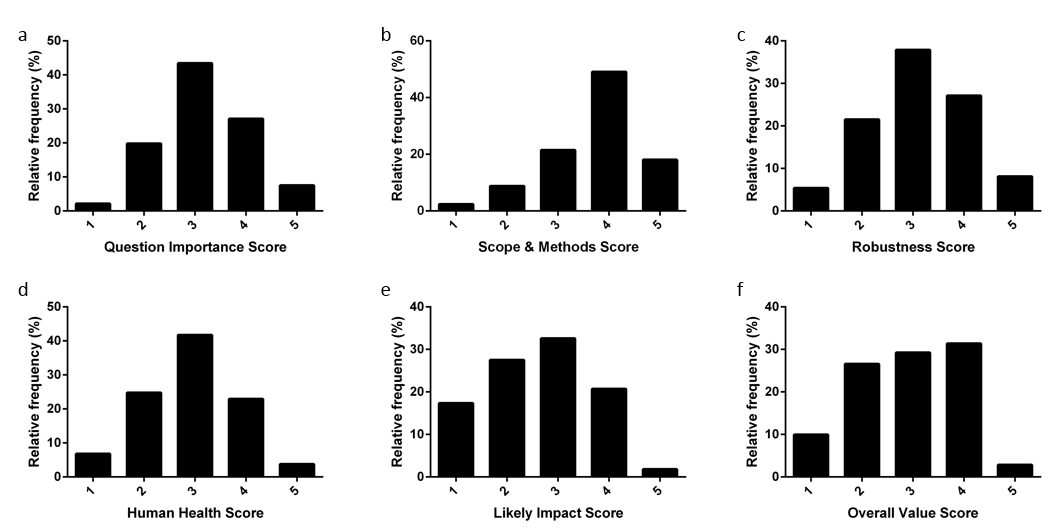

Supplement: S5 Fig — Distribution of ratings to the following questions: (A) rate whether the question being addressed is important to answer; (B) rate whether you agree that the methods are appropriate and the scope of the experiments adequate; (C) rate how robust the study is based on the strength of the evidence presented; (D) rate the likelihood that the results could ultimately have a substantial positive impact on human health outcomes; (E) rate the impact that the research is likely to have or has already had; and (F) provide your overall evaluation of the value and impact of this publication. (TIF) [file pbio.1002541.s005.tif]

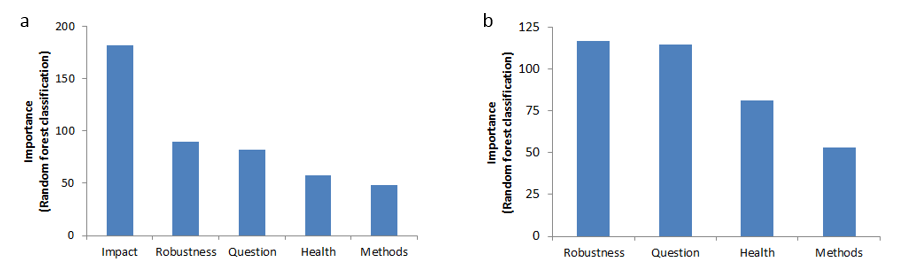

Supplement: S6 Fig — (A) Criteria most strongly linked to assessments of “overall value,” measured with Random Forest classification. Values indicate the mean decrease in Gini coefficient. (B) Criteria most strongly linked to assessments of “overall value,” excluding “likely impact,” and measured with Random Forest classification. (TIF) [file pbio.1002541.s006.tif]

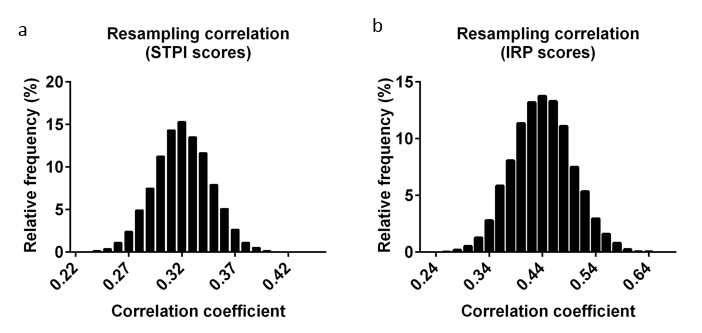

Supplement: S7 Fig — (A) Pearson correlation coefficients (r) of one randomly chosen reviewer score versus the mean of the other two scores for that paper, determined by statistical resampling. Distribution of correlation coefficients determined by resampling from the STPI dataset (10,000 repetitions, mean r = 0.32). (B) Pearson correlation coefficients (r) of one randomly chosen reviewer score versus the mean of the other two scores for that paper, determined by statistical resampling. Distribution of correlation coefficients determined by resampling from the IRP dataset (10,000 repetitions, mean r = 0.44). (TIF) [file pbio.1002541.s007.tif]

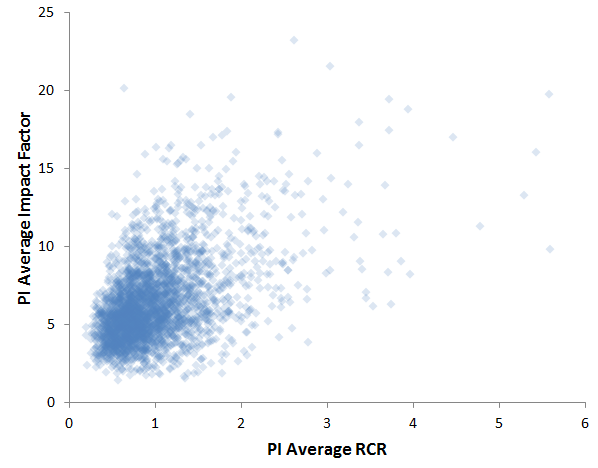

Supplement: S8 Fig — Some investigators published very influential articles (high RCR) in lower-profile venues (low JIF) and vice versa. R2 = 0.23. (TIF) [file pbio.1002541.s008.tif]

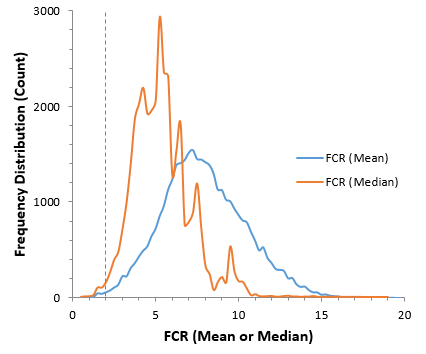

Supplement: S9 Fig — (TIF) [file pbio.1002541.s009.tif]

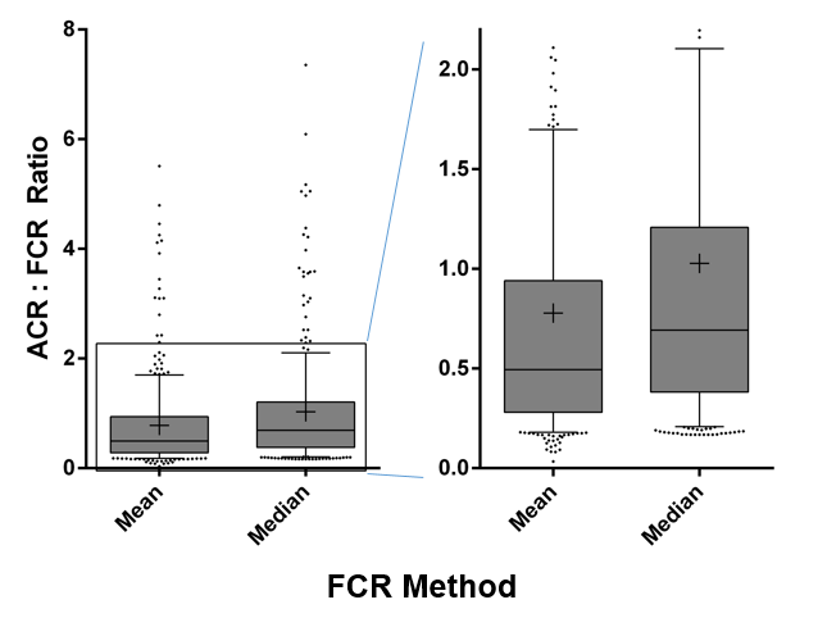

Supplement: S10 Fig — The method for aggregating the JCRs of the articles in the co-citation network was varied and the ACR:FCR ratios compared. (TIF) [file pbio.1002541.s010.tif]
