# Supplementary material for: Relative Citation Ratio (RCR): A New Metric That Uses Citation Rates to Measure Influence at the Article Level
Source: PLoS Biol. 2016 Sep 6;14(9):e1002541. doi: 10.1371/journal.pbio.1002541 (PMC5012559; doi:10.1371/journal.pbio.1002541)
Supplement: S1 Text — (DOCX) [file pbio.1002541.s018.docx]

Table of Contents

[Supporting Information 2](#_Toc451443218)

[Characterization of the co-citation networks of single articles 2](#_Toc451443219)

[Algorithm and calculations for Relative Citation Ratios (RCRs) 2](#_Toc451443220)

[Evaluation of different levels of the citation network for field normalization 5](#_Toc451443221)

[Validation of RCR with post-publication peer review 6](#_Toc451443222)

[Ranking invariance of RCR 9](#_Toc451443223)

[Susceptibility to gaming 11](#_Toc451443224)

[Effects of drifting fields over time 11](#_Toc451443225)

[Investigator-level bibliometrics over long periods 12](#_Toc451443226)

[Supporting References 13](#_Toc451443227)

# Supporting Information

## Characterization of the co-citation networks of single articles

Field normalization of citations is critical for cross-field comparisons, because of intrinsic differences in citation rates across disciplines. Our draws upon the idea of comparing the Article Citation Rate of an article (ACR) with an Expected Citation Rate (ECR), calculated based on peer performance in an article’s area of research [1,2]. Our Relative Citation Ratio should not be confused with a previously developed Relative Citation Rate [3]. Calculating a robust ECR is challenging; other methods frequently employ journals or journal categories as a proxy for a scientific field [4–10]. Unfortunately, these methods do not have sufficient precision to work well at the article level [11]. Because modern fields of biomedical research exist as a spectrum rather than discrete and separate fields [12], we decided to take a more nuanced approach to defining an article’s field. We constructed each article’s co-citation network [13] and used that as a representative sample of its area of research. Simply put, when an article is first cited, the other papers appearing in the reference list along with the article comprise its co-citation network (**Fig 1**). As the article continues to be cited, the papers appearing in the new reference lists alongside it are added to its co-citation network. This network provides a dynamic view of the article’s field of research, taking advantage of information provided by the experts who have found the study useful enough to cite.

## Algorithm and calculations for Relative Citation Ratios (RCRs)

Our algorithm uses the following steps to calculate RCR values, giving a ratio of ACR to ECR that is benchmarked to papers funded through NIH R01s:

1. Convert the RA citation counts to citations per year (**Fig 3** and **Supporting Equation S1**).
2. Generate the RA’s co-citation network. To do this, we assemble all articles citing the RA; the complete set of papers cited in the reference lists of these citing articles comprises the co-citation network (**Fig 1**).
3. Estimate the FCR of the RA by averaging the journal citation rates of the papers in the co-citation network (**Supporting Equation S3**).
4. Generate an ECR from the benchmark set of papers. Using R01-funded papers published in a given year, a linear regression of the ACRs vs. FCRs is performed (**Fig 3** and **Supporting Equations S3-4**). Regressions are calculated for each publication year.
   1. The linear equation coefficients corresponding to the RA’s publication year rescale its FCR into a denominator (ECR) that is benchmarked to the performance of R01-funded articles (**Supporting Equation S5**).
5. The Relative Citation Ratio is the ratio of the ACR : ECR.

When converting raw citation counts to Article Citation Rate ($Acr$), the year in which the RA was published was excluded from the denominator.

**Supporting Equation S1:**

$$Acr=\frac{Total citations to article}{Last year in citation database-Year of article publication}$$

We made this design decision because the publication year is nearly always partial, and because articles receive a low number of citations in the calendar year of their publication compared to subsequent years (**S2 Fig**). In practice, the sum of the citations in years 0 and 1 (the year of publication and the following year) is close to the mean number of citations per year in the following 8 years (**S2 Fig**).

ACRs and journal citation rates vary widely from field to field. To compare the Relative Citation Ratios of small groups (like individual investigators), special care must be taken to adjust only the fraction of the citation rate that is due to between-field differences. We tested three methods for adjusting expected citation rates to a field. These approaches each use information from an article’s citation network (see schematics in **Fig 1a**). The first method selects the reference article plus those cited in in its reference list (**Fig 1a**, bottom). The second approach instead selects subsequent papers citing the article (**Fig 1a**, top). Finally, the third uses the set of articles that are co-cited with the article of interest by subsequent papers (**Fig 1a**, middle). The Reference Article was always included in the set of papers selected to estimate the FCR, since an article is de facto part of its field. In all three cases, the average of the journal citation rates for the papers in the selected level of the citation network is used as the Field Citation Rate ($Fcr$).

**Supporting Equation S2:**

$$Fcr= \frac{\sum{Jcr}_{i}}{N}$$

$N$ is the number of papers in the selected level of the co-citation network (**Fig 1a**) and ${Jcr}_{i}$ is the journal citation rate of each paper at the specified level of the citation network.

We also investigated whether using the median ${Jcr}_{i}$ gives a better result than the arithmetic mean. During this process we found that using median normalization has at least three undesirable properties. First, it generates a non-normal distribution of FCR values (**S9 Fig**). While the mean-based method for calculating FCRs gives an approximately Gaussian distribution, the median-based method, in addition to a troublesome skew towards lower denominators, is unusual, displaying several peaks. Second, it disrupts field normalization, which is easily observed in an analysis of the articles of greatest concern, i.e. those with the lowest denominators (FCR < 2; dashed line in **S9 Fig**, and **S10 Fig**). Due to the skew towards lower denominators, it more than double the number of articles at the extreme low end of the FCR distribution (FCR < 2), which leads to an inflation of ACR/FCR ratios (**S10 Fig**) and the resulting RCR values. In other words, this alternative method has the undesirable effect of increasing the number of articles that have a high ratio because of a low denominator rather than a high numerator.

To generate an expectation of citation performance using this cohort of papers, we performed a linear regression of Article Citation Rate ($Acr$) in a baseline population against their Field Citation Rates ($Fcr$) from the same year (**Fig 3d**). R01-funded articles were used as a benchmark population. This process was repeated for each year being analyzed to give regression coefficients (slope, $\hat{B}$ and intercept,$\hat{a}$ ) for benchmarking articles.

**Supporting Equation S3:**

$$\hat{B}= \frac{\sum({Fcr}_{i}-\bar{Fcr}) ({Acr}_{i}-\bar{Acr})}{\sum{({Fcr}_{i}-\bar{Fcr})}^{2}}$$

**Supporting Equation S4:**

$$\hat{a}= \bar{Acr}- \hat{B} \times\bar{Fcr}$$

Alternatively, if benchmarking to the median (rather than mean) field-normalized performance for the benchmark group is desired, quantile regression can be used in lieu of simple linear regression [14]. In either case, the resulting regression line transforms a FCR into an Expected Citation Rate (${Ecr}^{Year}$), corresponding to the ACR that R01-funded papers with the same FCRs and published in the same year were able to achieve, and this can be used outside of the baseline population as a benchmark for articles published in that year:

**Supporting Equation S5:**

$${Ecr}^{Year}=\hat{B} \times Fcr+\hat{a}$$

For the years analyzed here (2002-2011), the resulting regression coefficients are given in S2 Table. RCR for each article is the ratio of that article’s ACR divided by its ECR. Calculating the arithmetic mean is the preferred way of determining the RCR of an entire portfolio [7–9]:

**Supporting Equation S6:**

$$RCR= \frac{1}{n}\sum\frac{{Acr}_{i}}{{Ecr}_{i}^{Year}}$$

Where $n$ is the number of papers being evaluated, ${Acr}_{i}$ is the article citation rate and ${Ecr}_{i}^{Year}$ the expected citation rate of each article found by transforming its ${Fcr}_{i}$ with the regression coefficients for its publication year (**Supporting Equation S5**).

## Evaluation of different levels of the citation network for field normalization

The aim of this adjustment is to accurately normalize an individual article’s citation rate to its field’s average citation rate, while preserving within-field differences between papers. Using a set of papers from 2009 matched to R01 grants active in the same year, we compared these three approaches. Calculated RCRs were very similar for all three groups at the article level (**S3 Table**). This is not surprising, since at this granular scale the article’s numerator (ACR) accounts for more of the variance than the denominator. However, accurate field-adjustment is especially important for large-scale analyses, where field differences in citation rates can dominate measurements, as article-level differences in ACR average out. A more accurate estimate of the field citation rate would be predicted to show a smaller correlation between article citation rate and field citation rate, as within-field differences are more effectively excluded. To measure the effectiveness of each level of the citation network, we calculated the correlations between ACRs and ECRs for each approach. Of the three, the “Co-cited” method shows the least correlation between article citations and expected citations (**S4 Table**). In addition, the variance in expected citation rates should be lower in approaches that more successfully isolate the between-field differences in citation rate from the within-field differences. Again, the co-citation level of the citation network performed the best here (**Table 1**).

## Validation of RCR with post-publication peer review

We extensively validated article-level RCRs against expert reviewer scores of the impact or value of papers, using post-publication peer review. Three independently collected sets of post-publication peer reviews were used for this analysis: Faculty of 1000 (F1000) [6,15], a previous survey conducted by the Institute for Defense Analyses Science and Technology Policy Institute (STPI) [16], and post-publication peer review conducted by NIH Intramural Research Program (IRP) Principal Investigators.

The first set of expert review scores was compiled from F1000, in which faculty review articles in their fields of expertise, and rate the articles on a scale of 1 to 3 (“Good”, “Very Good”, and “Exceptional”). Because the decision by the faculty members to review the article is itself a mark of merit, these scores are summed into a composite F1000 score (**S3 Fig**). We downloaded scores in June 2014 for 2193 R01-funded articles published in 2009 and compared them to their RCRs. This yielded an article-level correlation coefficient ***r*** of 0.44 between RCR and F1000 scores (**Fig 4a**).

For a second set of expert review scores, we took advantage of a previous survey conducted by STPI, of papers funded through the Howard Hughes Medical Institute and NIH. In this survey, experts rated the impact of articles (shown here on a scale of 0 to 4, n = 430 papers from 2005-2011, **S4 Fig**). Since citation data (including RCR) is highly skewed while survey ratings are not, RCR was log-transformed to bring these ranges into better alignment. The article-level correspondence of RCR with these review scores was similar to that observed with the F1000 scores (***r*** = 0.47, **Fig 4b**).

Finally, we recruited investigators from the NIH Intramural Research Program (IRP) to perform post-publication peer review of NIH-funded articles published in 2009 (**S5 Fig, S6 Fig, S7 Fig**). A total of 290 articles were independently reviewed by multiple investigators, yielding an article-level correlation of 0.56 (**Fig 4c**). The distribution of these impact scores is shown in **S5 Fig**. Finally, we asked reviewers in the NIH Intramural Research Program to conduct post-publication peer review of R01-funded articles published in 2009 (reviews conducted by The Scientific Consulting Group, Gaithersburg, MD). Reviewers were asked to give scores on a scale of 1-5 for the following questions:

- Rate whether the question being addressed is important to answer. (1 = Not Important, 2 = Slightly Important, 3 = Important, 4 = Highly Important, 5 = Extremely Important)
- Rate whether you agree that the methods are appropriate and the scope of the experiments adequate. (1 = Strongly Disagree, 2 = Disagree, 3 = Neutral, 4 = Agree, 5 = Strongly Agree)
- Rate how robust the study is based on the strength of the evidence presented. (1 = Not Robust, 2 = Slightly Robust, 3 = Moderately Robust, 4 = Highly Robust, 5 = Extremely Robust)
- Rate the likelihood that the results could ultimately have a substantial positive impact on human health outcomes. (1 = Very unlikely, 2 = Unlikely, 3 = Foreseeable but uncertain, 4 = Probable, 5 = Almost Certainly)
- Rate the impact that the research is likely to have or has already had. (1 = Minimal Impact, 2 = Some Impact, 3 = Moderate Impact, 4 = High Impact, 5 = Extremely High Impact)
- Provide your overall evaluation of the value and impact of this publication. (1 = minimal or no value, 2 = Moderate value, 3 = Average value, 4 = High value, 5= Extremely high value)

The distribution of responses for each of these questions is shown in **S5 Fig**. Multiple experts were asked to review each paper, and each set of papers were matched to the fields of expertise of the reviewers examining them. For correlating RCR to review scores, we used the average of the score for the final question (overall evaluation of the paper’s value) for articles that were reviewed by at least two experts.

To determine post-hoc which of the first five criteria (importance of scientific question, appropriate methods, robustness of study, likelihood of health outcomes and likely impact) were associated with reviewers’ ratings of overall value, we first performed a Random Forest analysis using all 5 criteria. Perhaps unsurprisingly, this analysis showed that “likely impact” was most closely associated with assessment of overall value (**S6 Fig**). We subsequently removed this criterion from the analysis to determine the relative importance of the other 4 questions. In this analysis, “Importance” of the scientific question and “Robustness” were most closely linked to overall value (**S6 Fig**).

Should an article-level correlation of approximately 0.5 between RCR and expert reviewer scores be considered reliable? This level of correspondence is similar to that previously measured between bibliometric indicators and reviewer scores [6,15]. Given the partially overlapping datasets used here, we were able to calculate the correlation of expert reviewer scores with one another. The Pearson correlation coefficient between log-transformed F1000 scores and those from the STPI review was 0.35. In addition, the correlation of scores within a survey can be determined with statistical resampling. We selected papers with three reviews from the STPI and NIH IRP surveys. The order of reviewers was randomly shuffled, and the correlation coefficient between the first reviewer’s score and the mean of the other two scores was determined and recorded. This process was repeated 10,000 times for each dataset. The distributions of the 10,000 recorded correlation coefficients are shown in **S7 Fig**. This approach demonstrated an internal correlation of 0.32 for STPI reviews and 0.44 for the NIH IRP reviews. These values are similar to the correlation between RCR and each set of review scores. These internal correlations between reviewer scores likely represent an estimate of the degree to which it is possible for bibliometrics to correspond to expert opinions. Thus, RCR agrees with expert opinion scores as well as experts agree with one another.

## Ranking invariance of RCR

One desirable property in a bibliometric indicator is that of ranking invariance [9,17,18]. A citation metric is ranking invariant if, when two groups of articles are being compared using that indicator, their relative ranking does not change if the groups are inflated by the same amount of uncited papers [18]. RCR is ranking invariant under two cases: when the two comparison groups are the same size and the same absolute number of uncited papers is added to each group, and when the two comparison groups are different sizes and the same proportion of uncited papers is added to each comparison group.

For the first case (two groups of the same size, termed groups $I$ and $J$, where group $I$ has the greater RCR), the group RCRs are described by the following inequality:

**Supporting Equation S7:**

$$\frac{\sum_{1}^{n} \frac{{Acr}_{i}}{{Ecr}_{i}}}{n}> \frac{\sum_{1}^{n} \frac{{Acr}_{j}}{{Ecr}_{j}}}{n}$$

Where $i$ and $j$ are the individual papers from groups $I$ and $J$, and $n$ is the number of papers in these groups. Adding $k>0$ papers to each group, each with a constant RCR of $a \geq0$ (equal to 0 for uncited papers) yields the following inequality:

**Supporting Equation S8:**

$$\frac{\left( \sum_{1}^{n} \frac{{Acr}_{i}}{{Ecr}_{i}} \right)+ak}{n+k}> \frac{\left( \sum_{1}^{n} \frac{{Acr}_{j}}{{Ecr}_{j}} \right)+ak}{n+k}$$

This simplifies to Supporting Equation S9, demonstrating ranking invariance under this condition:

**Supporting Equation S9:**

$$\sum_{1}^{n} \frac{{Acr}_{i}}{{Ecr}_{i}}> \sum_{1}^{n} \frac{{Acr}_{j}}{{Ecr}_{j}}$$

For the second case (two groups of unequal sizes, termed groups $I$ and $J$, where group $I$ has the greater RCR), the group RCRs are described by the following inequality:

**Supporting Equation S10:**

$$\frac{\sum_{1}^{n} \frac{{Acr}_{i}}{{Ecr}_{i}}}{n}> \frac{\sum_{1}^{n} \frac{{Acr}_{j}}{{Ecr}_{j}}}{m}$$

Where $n$ is the number of papers in group $I$ and $m$ is the number of papers in group $J$. Adding the same proportion $k>0$ of papers to each group, each with a constant RCR of $a\geq0$ (equal to 0 for uncited papers) yields the following inequality:

**Supporting Equation S11:**

$$\frac{\left( \sum_{1}^{n} \frac{{Acr}_{i}}{{Ecr}_{i}} \right)+akn}{n\left( 1+k \right)}> \frac{\left( \sum_{1}^{n} \frac{{Acr}_{j}}{{Ecr}_{j}} \right)+akm}{m\left( 1+k \right)}$$

This simplifies back to **Supporting Equation S10**, demonstrating ranking invariance under this condition as well. Note that while uncited papers correspond $a=0$, any positive RCR $a$ could be substituted and ranking invariance would hold.

## Susceptibility to gaming

Since willingness to game Impact Factors is so prevalent [19,20], it stands to reason that some researchers may be tempted to game their RCRs. Self-citation seems to be the most obvious route for boosting the numerator, which is a limitation for citation metrics in general. Is RCR susceptible to gaming of the denominator? Consider this thought experiment: a researcher under career pressure seeks to boost the RCR of one of his papers by lowering its denominator. Is this feasible? In an extreme example, he may publish a new paper citing his previous article, along with 40 others in journals with Impact Factors of 1.0. Effects for a real article published in 2008 with an RCR close to 1.0 are shown in **S5 Table**. The effect of this egregious example is equivalent to only a single additional citation, indicating that even obvious attempts to weigh the co-citation network have, at best, a marginal effect. Obviously, newer articles would be more susceptible to manipulation, but it is unlikely that a researcher would be willing to so overtly attempt to game the metric; much more likely is an attempt to preferentially co-cite related articles in journals at the lower end of normal for the researcher's field (but not an order of magnitude like our thought experiment). This less obvious form of manipulation would reward the equivalent of much less than one additional citation, and would not have much of an impact.

## Effects of drifting fields over time

Imagine a field whose citation rate drifted significantly over the course of a decade. This field’s intrinsic citation rate went from 6 to 4 over the course of 10 years, and new citations to previously published articles declined by the same amount, in keeping with the field. In the example in **S6 Table**, yearly citations (“Cites”) and their contributions toward FCR (“FCR (Yr.)”) were added as the field’s citation rate declined, contributing lower values to FCR as time went on. The changes in CPY and FCR are shown as separate columns; because these are based on cumulative metrics, their decline lags behind the field as a whole. New article contributions to the co-citation network were modeled with a linear relationship based on empirical results (**Fig 1**). Despite the substantial 33% reduction in the citation rates to this article and its field, the ratio between CPY and FCR is nearly unchanged.

## Investigator-level bibliometrics over long periods

To test the degree to which investigator-level metrics, as shown in **Fig 8** and **Table 2**, are stable over long periods, the analysis was repeated with the 2002-2014 dataset used for the Field Citation Rate stability analysis in **Fig 3**. As in **Fig 8**, investigators with continual R01 funding through the entire window (2002-2013) were selected for inclusion, to rule out additional variance from a catastrophic loss of funding. Rather than comparing two adjacent 4-year windows, two 2-year windows (2002-2003 and 2012-2013) were used spanning a decade. Correlations comparing these two periods are shown in **S7 Table**. Investigator-level correlations are present at this longer time frame, although as expected over a longer time window, at a somewhat lower level. Because this analysis uses two 2-year windows rather than two 4-year windows, this result may underestimate the size of the effect compared to **Fig 8** and **Table 2**.

# Supporting References

1. Schubert A, Glänzel W, Braun T (1986) Relative indicators of publication output and citation impact of european physics research, 1978–1980. Czechoslov J Phys 36: 126–129. doi:10.1007/BF01599744.

2. Vinkler P (2003) Relations of relative scientometric indicators. Scientometrics 58: 687–694. doi:10.1023/B:SCIE.0000006888.69146.24.

3. Schubert A, Braun T (1986) Relative indicators and relational charts for comparative assessment of publication output and citation impact. Scientometrics 9: 281–291. doi:10.1007/BF02017249.

4. Moed HF, Burger WJM, Frankfort JG, Van Raan AFJ (1985) The use of bibliometric data for the measurement of university research performance. Res Policy 14: 131–149. doi:10.1016/0048-7333(85)90012-5.

5. Zitt M, Small H (2008) Modifying the journal impact factor by fractional citation weighting: The audience factor. J Am Soc Inf Sci Technol 59: 1856–1860. doi:10.1002/asi.20880.

6. Bornmann L, Leydesdorff L (2013) The validation of (advanced) bibliometric indicators through peer assessments: A comparative study using data from InCites and F1000. J Informetr 7: 286–291. doi:10.1016/j.joi.2012.12.003.

7. Opthof T, Leydesdorff L (2010) Caveats for the journal and field normalizations in the CWTS (“Leiden”) evaluations of research performance. J Informetr 4: 423–430. doi:10.1016/j.joi.2010.02.003.

8. van Raan AFJ, van Leeuwen TN, Visser MS, van Eck NJ, Waltman L (2010) Rivals for the crown: Reply to Opthof and Leydesdorff. J Informetr 4: 431–435. doi:10.1016/j.joi.2010.03.008.

9. Waltman L, van Eck NJ, van Leeuwen TN, Visser MS, van Raan AFJ (2011) Towards a new crown indicator: Some theoretical considerations. J Informetr 5: 37–47. doi:10.1016/j.joi.2010.08.001.

10. Lundberg J (2007) Lifting the crown—citation z-score. J Informetr 1: 145–154. doi:10.1016/j.joi.2006.09.007.

11. Leydesdorff L, Bornmann L (2015) The operationalization of “fields” as WoS subject categories (WCs) in evaluative bibliometrics: The cases of “library and information science” and “science & technology studies.” J Assoc Inf Sci Technol: n/a – n/a. doi:10.1002/asi.23408.

12. Talley EM, Newman D, Mimno D, Herr BW, Wallach HM, et al. (2011) Database of NIH grants using machine-learned categories and graphical clustering. Nat Methods 8: 443–444. doi:10.1038/nmeth.1619.

13. Small H (1973) Co-citation in the scientific literature: A new measure of the relationship between two documents. J Am Soc Inf Sci 24: 265–269. doi:10.1002/asi.4630240406.

14. Koenker R (2005) Quantile Regression. Cambridge University Press. 349 p.

15. Li X, Thelwall M (2012) F1000, Mendeley and Traditional Bibliometric Indicators. Proc 17th Int Conf Sci Technol Indic Montréal Sci OST 3: 541–551.

16. Lal B, Wilson AG, Jonas S, Lee EC, Richards AM, et al. (2012) An Outcome Evaluation of the National Institutes of Health (NIH) Director’s Pioneer Award (NDPA) Program, FY 2004–2006.

17. Glänzel W, Moed HF (2012) Opinion paper: thoughts and facts on bibliometric indicators. Scientometrics 96: 381–394. doi:10.1007/s11192-012-0898-z.

18. Rousseau R, Leydesdorff L (2011) Simple arithmetic versus intuitive understanding:The case of the impact factor. ISSI Newsl: 10–14.

19. Wilhite AW, Fong EA (2012) Scientific publications. Coercive citation in academic publishing. Science 335: 542–543. doi:10.1126/science.1212540.

20. Krell F-T (2010) Should editors influence journal impact factors? Learn Publ 23: 59–62. doi:10.1087/20100110.
